# Copper(II) import and reduction are dependent on His-Met clusters in the extracellular amino terminus of human copper transporter-1

**DOI:** 10.1016/j.jbc.2022.101631

**Published:** 2022-01-26

**Authors:** Sumanta Kar, Samarpita Sen, Saptarshi Maji, Deepashri Saraf, Ruturaj, Rupam Paul, Sohini Dutt, Basudeb Mondal, Enrique Rodriguez-Boulan, Ryan Schreiner, Durba Sengupta, Arnab Gupta

**Affiliations:** 1 Department of Biological Sciences, Indian Institute of Science Education and Research Kolkata, Mohanpur, West Bengal, India; 2 Physical and Materials Chemistry Division, CSIR-National Chemical Laboratory, Pune, Maharashtra, India; 3 Department of Chemical Sciences, Indian Institute of Science Education and Research Kolkata, Mohanpur, West Bengal, India; 4 Department of Ophthalmology, Margaret Dyson Vision Research Institute, Weill Cornell Medicine, New York, New York, USA

**Keywords:** CTR1, SLC31A1, copper homeostasis, polarized epithelia, MDCK, BSA, bovine serum albumin, CRE, common recycling endosome, CTR1, copper transporter-1, DFT, density functional theory, DMEM, Dulbecco’s modified eagle’s medium, EPR, electronic paramagnetic resonance, FBS, fetal bovine serum, hCTR1, human CTR1, HSA, human serum albumin, ICP-OES, inductively coupled plasma optical emission spectroscopy, MDCK, Madin Darby Canine Kidney, RPE, retinal pigment epithelium, TM, transmembrane

## Abstract

Copper(I) is an essential metal for all life forms. Though Cu(II) is the most abundant and stable state, its reduction to Cu(I) *via* an unclear mechanism is prerequisite for its bioutilization. In eukaryotes, the copper transporter-1 (CTR1) is the primary high-affinity copper importer, although its mechanism and role in Cu(II) reduction remain uncharacterized. Here we show that extracellular amino-terminus of human CTR1 contains two methionine-histidine clusters and neighboring aspartates that distinctly bind Cu(I) and Cu(II) preceding its import. We determined that hCTR1 localizes at the basolateral membrane of polarized MDCK-II cells and that its endocytosis to Common-Recycling-Endosomes is regulated by reduction of Cu(II) to Cu(I) and subsequent Cu(I) coordination by the methionine cluster. We demonstrate the transient binding of both Cu(II) and Cu(I) during the reduction process is facilitated by aspartates that also act as another crucial determinant of hCTR1 endocytosis. Mutating the first Methionine cluster (^7^Met-Gly-Met^9^) and Asp^13^ abrogated copper uptake and endocytosis upon copper treatment. This phenotype could be reverted by treating the cells with reduced and nonreoxidizable Cu(I). We show that histidine clusters, on other hand, bind Cu(II) and are crucial for hCTR1 functioning at limiting copper. Finally, we show that two N-terminal His-Met-Asp clusters exhibit functional complementarity, as the second cluster is sufficient to preserve copper-induced CTR1 endocytosis upon complete deletion of the first cluster. We propose a novel and detailed mechanism by which the two His-Met-Asp residues of hCTR1 amino-terminus not only bind copper, but also maintain its reduced state, crucial for intracellular uptake.

Copper is a micronutrient essential for all eukaryotic organisms as it plays a crucial role in coordinating different physiological activities of cells (1, 2, 3, 4). Copper shuttles between its two primary oxidation states, Cu(I) and Cu(II). Though it participates in physiological processes, the cuprous ion Cu(I) is unstable in the oxidizing environment; Cu(II) is the most abundant oxidation state in hydrophilic and oxidizing environments (5). In yeasts, copper is first reduced from Cu(II) to Cu(I) by cell surface reductases Fre1/Fre2, prior to uptake (6, 7, 8). However, the mechanism of Cu(II) reduction in mammalian cells is not clearly understood. In this study, we, for the first time, show that the human copper transporter-1 (hCTR1), besides importing copper, also plays a crucial role in maintaining the Cu(I) redox state that renders the metal bioavailable for physiological utilization in cells.

CTR1 (SLC31A1) is the only high-affinity plasma membrane copper importer that has been known to date in mammalian cells (9, 10). CTR1 is the primary member of the CTR family consisting of six known members (CTR1–6), with at least one member found in all eukaryotic life forms (11). The hCTR1 is a small protein of 21 kDa, consisting of 190 amino acids. It exists as a homotrimeric integral membrane protein with each monomer consisting of an extracellular amino-terminal (N-terminus), three transmembrane (TM) domains, and a small intracellular cytosolic tail (12, 13, 14). The extracellular N-term shows high sequence variability among species. hCTR1 N-terminus is 67 amino acids long and is predicted to be unstructured (14). The amino terminus of hCTR1 also harbors N-linked (Asn^15^) and O-linked glycosylation (Thr^27^). O-linked glycosylation at Thr-27 is necessary to prevent proteolytic cleavage that removes approximately half of the N-terminus of hCTR1 (15).

In nonpolarized epithelial cells, *e.g.*, HEK293T, hCTR1 localizes on the plasma membrane in basal or copper-limiting conditions (16). In high-copper treatments, as a self-regulatory mechanism to limit copper import, hCTR1 endocytoses in vesicles and accumulates in early sorting and recycling endosomes marked by Rab5 and EEA1 (17). Using live-cell imaging, Clifford *et al.* (17) demonstrated that, upon removal of extracellular copper, hCTR1 recycles back to the plasma membrane through the Rab11-dependent pathway. Using another nonpolarized model, *i.e.*, HeLa cells, Curnock and Cullen have further shown that the retromer complex regulates plasma membrane recycling of the protein and prevents it from entering the lysosomal degradation pathway (18). However, localization of hCTR1 in polarized epithelia has been a field of debate with several contrasting reports. Data from Thiele group have shown that CTR1 localizes at the apical membrane in mouse intestine and is responsible for luminal copper uptake (19, 20). On the other hand, Zimnicka *et al.* (21) demonstrated that CTR1 localizes on the basolateral surface of kidney epithelial cell model, Madin Darby Canine Kidney (MDCK), enterocyte models Caco-2, as well as a model for intestinal crypt cells, T84. A clearer understanding of hCTR1 localization in the polarized epithelial cell is warranted as its crucial extracellular N-terminal domain would be exposed to two completely different extracellular environments at luminal (apical) *versus* blood (basolateral) sides that will possibly influence copper availability, copper binding, and uptake by the protein. However, hCTR1 might exhibit differential localization on apical *versus* basolateral membrane directed by tissue type and the origin of the polarized cell line used in the experiment. For instance, the α subunit of the Na,K ATPase is localized on the basolateral surface of most polarized epithelial cells, but is targeted on the apical membrane of the retinal pigment epithelium (RPE) (22). The human LDL receptor is found on the apical surface in kidney tubules and on basolateral surface of intestinal cells (http://rupress.org/jcb/article-pdf/111/2/347/1255876/347.pdf, accessed December 2, 2021).

*In vitro* as well as *in vivo* studies have shown that the N-terminal domain can acquire copper from human serum albumin (HSA), one of the main Cu(II) carriers in the blood (23, 24, 25). The N-terminus domain of hCTR1 has several methionine and histidine-rich clusters that were previously shown to be essential for copper acquisition and copper binding (25, 26, 27). The presence of the **A**mino **T**erminal **Cu**(II)- and **N**i(II) binding site (ATCUN) spanning the first three amino acid residues of hCTR1 and characterized by the general sequence (H_2_N-Xaa-Zaa-His) favors a possibly direct Cu(II) transfer from HSA to hCTR1 amino-terminus (24, 28). Stefaniak *et al.* (25) have suggested that other residues adjacent to the ATCUN, namely His^5^, His^6^, and Asp^13^, might also be involved in Cu(II) binding along with the ATCUN cluster at a pH characteristic of the extracellular space. Intracellular copper exists primarily in reduced form, Cu(I), whereas in the extracellular environment, it is found in its higher oxidation state, Cu(II) (29). It has been hypothesized that extracellular reducing factor such as ascorbate and STEAP reductases might be responsible for Cu(II) reduction (30). Though *in vitro* studies from Haas group have shown that purified N-terminus of hCTR1 has the capacity to reduce Cu(II) in the presence of ascorbate; any direct role of N-terminus of hCTR1 in this reduction process and subsequent import *in vivo* is still speculative (31, 32). This N-terminal extracellular domain contains multiple methionines (M^7^GM^9^, the first Met-cluster and ^40^MMMMPM^45^, the second Met cluster) and histidine (H^3^-H^6^, H^22^-H^24^, and H^31^-H^33^) rich clusters that are possible anchoring sites for Cu(I) and Cu(II) ions, respectively. After shuttling through the N-terminus, the reduced copper is then passed through a Cu(I) specific selectivity filter formed by a conserved ^150^MXXXM^154^ sequence in the second TM domain before it is delivered to cytoplasmic copper chaperone proteins, such as Atox1 and CCS (33, 34).

Using a combination of computational and in-cell experimental techniques, we postulate a model that correlates and links the three main functionalities of the protein, *i.e.*, (a) distinct Cu(I) and Cu(II) binding to hCTR1 N-terminus, (b) Cu(I) import, and finally, (c) hCTR1 endocytosis. We highlight the mechanism by which hCTR1 N-terminal methionine clusters in association with the aspartates maintain the reduced redox state of copper, hence rendering it bioavailable without cofactor requirements.

## Results

### hCTR1 facilitates copper uptake at the basolateral membrane in polarized epithelial cells

Due to its unique nature, mammalian polarized epithelial cells connect as well as partition the luminal side (apical) and blood side (basolateral) of the epithelial tissue. Copper is required to be imported in cells from either blood plasma or from luminal contents of the epithelia for its systemic and intracellular utilization.

**Figure 1:**
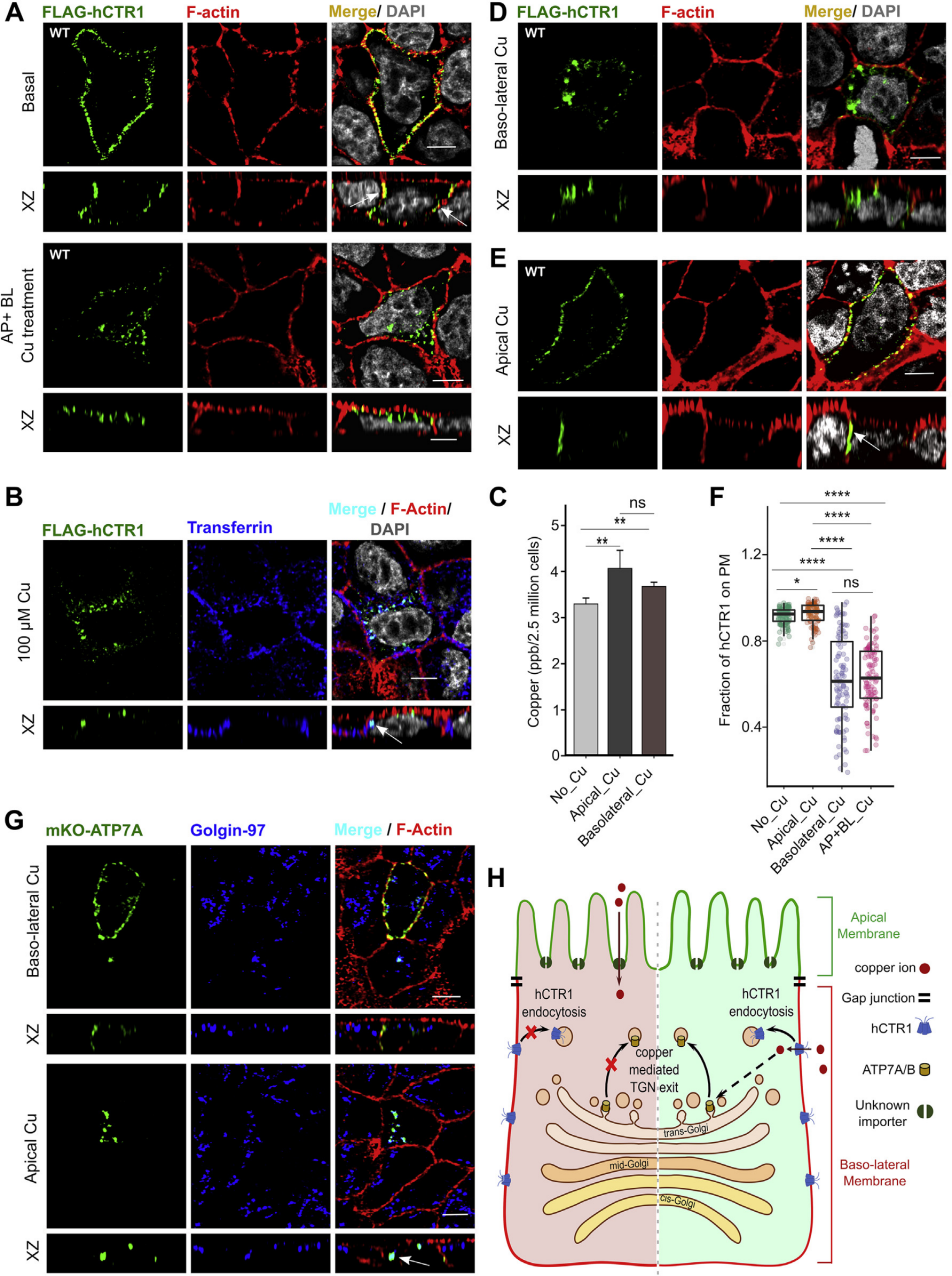
**Copper treatment induces hCTR1 endocytosis.***A*, wild-type (WT) FLAG-hCTR1 (*green*) under basal condition colocalizes with Phalloidin (*red*), white arrow in the XZ section exhibits basolateral localization of hCTR1 (*Upper panel*). WT FLAG-hCTR1 (*green*) endocytosed upon copper treatment from both the apical (AP) and basolateral (BL) sides of the cells (*Lower panel*). In the merged figures DAPI (*gray*) staining indicates nucleus. *B*, following copper (100 μM) mediated endocytosis, WT FLAG-hCTR1 colocalizes with the basolateral sorting endosomes and common recycling endosomes marked with post-30 min internalization of Transferrin-633 (*Blue*). In the merged figure DAPI (*gray*) staining indicates nucleus. *C*, comparison of copper uptake in polarized MDCK-II cells (n = 9) under different treatment conditions shows similar copper accumulation in case of both apical and basolateral copper treatment, copper concentrations were measured in parts per billion (ppb, mean ± SD)∗∗*p* < 0.01; ns, not significant (nonparametric Mann–Whitney U test/Wilcoxon rank-sum test). *D*, WT FLAG-hCTR1 (*green*) endocytoses in response to copper treatment only on the basolateral side of the cells. In the merged figures DAPI (*gray*) staining indicates nucleus. *E*, WT FLAG-hCTR1 (*green*) treated with copper on the apical side fails to endocytose and localizes at basolateral membrane. *F*, fraction of hCTR1 colocalization with membrane marker F-actin, demonstrated by a box plot with jitter points. The box represents the 25 to 75th percentiles, and the median in the middle. The whiskers show the data points within the range of 1.5 × interquartile range (IQR) from the first and third quartile. ∗*p* < 0.05, ∗∗∗∗*p* < 0.0001; ns, not significant (nonparametric Mann–Whitney U test/Wilcoxon rank-sum test). Sample size (n) for Basal: 100, AP: 91, BL: 101, AP+BL: 101. *G*, stably expressed mKO-ATP7A (*green*) in response to copper treatment at the bottom transwell chamber (*upper panel*) traffics to the basolateral membrane, whereas upon copper treatment at the top transwell chamber (*lower panel*), it localizes with Golgin97 (*blue*). *H*, schematic showing the physiological effects of apical *versus* basolateral copper entry, the latter through hCTR1. [In all the conditions, cells are polarized MDCK-II, XZ section shows the orthogonal sections of all the stacks, *green*- FLAG-hCTR1 and mKO-ATP7A separately, *red*: F-actin and *Blue*: Transferrin and Golgin-97 separately; scale bar: 5 μm]. hCTR1, human copper transporter-1; MDCK, Madin Darby Canine Kidney.

We used the well-characterized model of polarized epithelia, Madin Darby Canine Kidney (MDCK-II) cells to determine the effect of copper on localization of hCTR1 (35, 36). To determine the localization of hCTR1 in polarized epithelia, we transfected MDCK cells with Flag-*hCTR1*, seeded them on Transwell^R^ chambers, and allowed them to polarize until apical and basolateral plasma membrane domains were formed (Fig. S1, *A* and *B*). We used confocal microscopy to study the establishment of polarity using cortical actin and podocalyxin (gp-135) as a marker (Fig. S1*A*). Upon imaging the cells along the axial plane, we found that hCTR1 localizes completely on the basolateral surface with no signal on the apical membrane (Fig. 1*A*, *top panel*). Flag-hCTR1 also colocalized with Na,K-ATPase confirming its targeting on the basolateral surface (Fig. S1*C*). To ensure that the N-terminally tagged Flag sequence did not influence the targeting of the protein on the basolateral surface, we used a Myc-tagged *hCTR1* where the tag was inserted in the intracellular loop between the transmembranes 1 and 2 (between Ile^115^ and Leu^116^). The Myc-tagged protein also targeted normally at the basolateral membrane validating the use of the Flag-hCTR1 for subsequent experiments (Fig. S1*D*). Upon copper treatment (100 μM; 1 h), Flag-hCTR1 endocytoses to vesicular compartments (Fig. 1*A*, *bottom panel*). Dose–response studies established that 100 μM was the optimum copper concentration that triggers complete endocytosis of hCTR1 from plasma membrane to vesicles (Fig. S1*E*). Using pulsed transferrin (Tf) uptake assay (5 min and 30 min), we determined the identity of the endosomal compartments that harbors endocytosed hCTR1. We found that endocytosed hCTR1 primarily localizes at basolateral sorting endosomes (BSE) and the supranuclear common recycling endosomes (CRE) compartments marked with Tf post-30 min uptake (Fig. 1*B*). Previous studies have demonstrated that upon copper-induced endocytosis, a cleavage mediated by Cathepsin-B occurs at the amino terminus hCTR1 (37, 38). Since, the Flag-tag is on the extreme N-terminal end, it would be lost and not recognized once that cleavage happens after the protein is endocytosed and is ready for recycling to plasma membrane. So we used a double-tagged construct where Flag-tag was on the extreme N-terminal end, and Myc-tag was engineered between the transmembranes 1 and 2 on the cytosolic side of the protein. The Flag-Myc-hCTR1 was targeted normally to the basolateral membrane (Fig. S1*F*, upper panel). Upon copper treatment, it exhibited endocytosis. Upon staining with anti-Flag (green) and anti-Myc (red) antibodies, we detected signals from both Flag and Myc tags (merged as yellow) in most of the endocytosed hCTR1 (Fig. S1*F*, bottom panel). However, in some hCTR1 vesicles, we observed distinct green and red signals that possibly represent unprocessed (uncleaved) hCTR1 and N-terminal cleaved protein, respectively, which lack the Flag sequence but contained the Myc tag.

Apart from hCTR1, the divalent metal transporter DMT1 has been implicated as another copper importer, albeit at a low affinity, but at the apical membrane of polarized epithelia (39). Hence, we determined whether uptake of copper happens primarily through the apical or basolateral side of MDCK cells. Cells were polarized on Transwells and treated with 100 μM copper, added to either basolateral (bottom chamber) or apical side (top chamber). Using ICP-OES, we measured intracellular copper levels and found that luminal (apical) as well as basolateral membranes can uptake copper with similar efficacies (Fig. 1*C*). To determine whether hCTR1 endocytosis is triggered primarily in response to apically or basolaterally imported copper, we treated polarized MDCK cells expressing Flag-hCTR1 either in the top or bottom chamber of the transwells. We observed that upon copper treatment at the bottom chamber, hCTR1 endocytosed (Fig. 1*D*), whereas it continued to localize on the basolateral membrane upon copper treatment at the top chamber (Fig. 1*E*). Quantitation of colocalization between Flag-hCTR1 and F-Actin upon copper treatment at basolateral *versus.* apical sides of MDCK-II is illustrated in Figure 1*F*. To summarize, copper imported through the basolateral membrane triggers hCTR1 endocytosis.

Copper uptake at the basolateral membrane is mediated by hCTR1 and at the apical side by an unknown mechanism or possibly by DMT1 (10, 40). We used CuCl_2_ as the source of copper. Cu(II) needs to be reduced to bioavailable Cu(I) prior to it being utilized by intracellular proteins. We further investigated if both these copper pools (luminally *versus* basolaterally uptaken) are equally bioavailable and able to trigger physiological response inside the cell. We utilized copper-induced trafficking of the Copper-ATPases ATP7A and ATP7B from the trans-Golgi network to secretory vesicles as a readout of bioavailable or utilizable copper. It has been established that upon copper entry *via* hCTR1, copper is sequestered by a metallochaperone, Atox1. Atox1 delivers the copper to Copper-ATPases that traffic to vesicles to export out excess copper (41). Upon copper treatment at the basolateral side of MDCK-II, we observe TGN exit, vesicularization, and plasma membrane targeted trafficking of the ATP7A (Fig. 1*G*, *top panel*) as well as ectopically expressed ATP7B (Fig. S1*G*, *top panel*). However, copper treatment at the apical side, though leads to intracellular copper uptake, did not elicit any trafficking response of the Copper-ATPases, ATP7A (Fig. 1*G*, *bottom panels)* or ATP7B (Fig. S1*G*, *bottom panel*). In conclusion, copper that enters through the basolateral membrane *via* hCTR1 is bioavailable and therefore can evoke normal physiological response. A scheme summarizing the different physiological responses elicited by apical *versus* basolateral copper uptake is shown in Figure 1*H*.

### N-terminal of hCTR1 is critical for its plasma membrane localization

**Figure 2:**
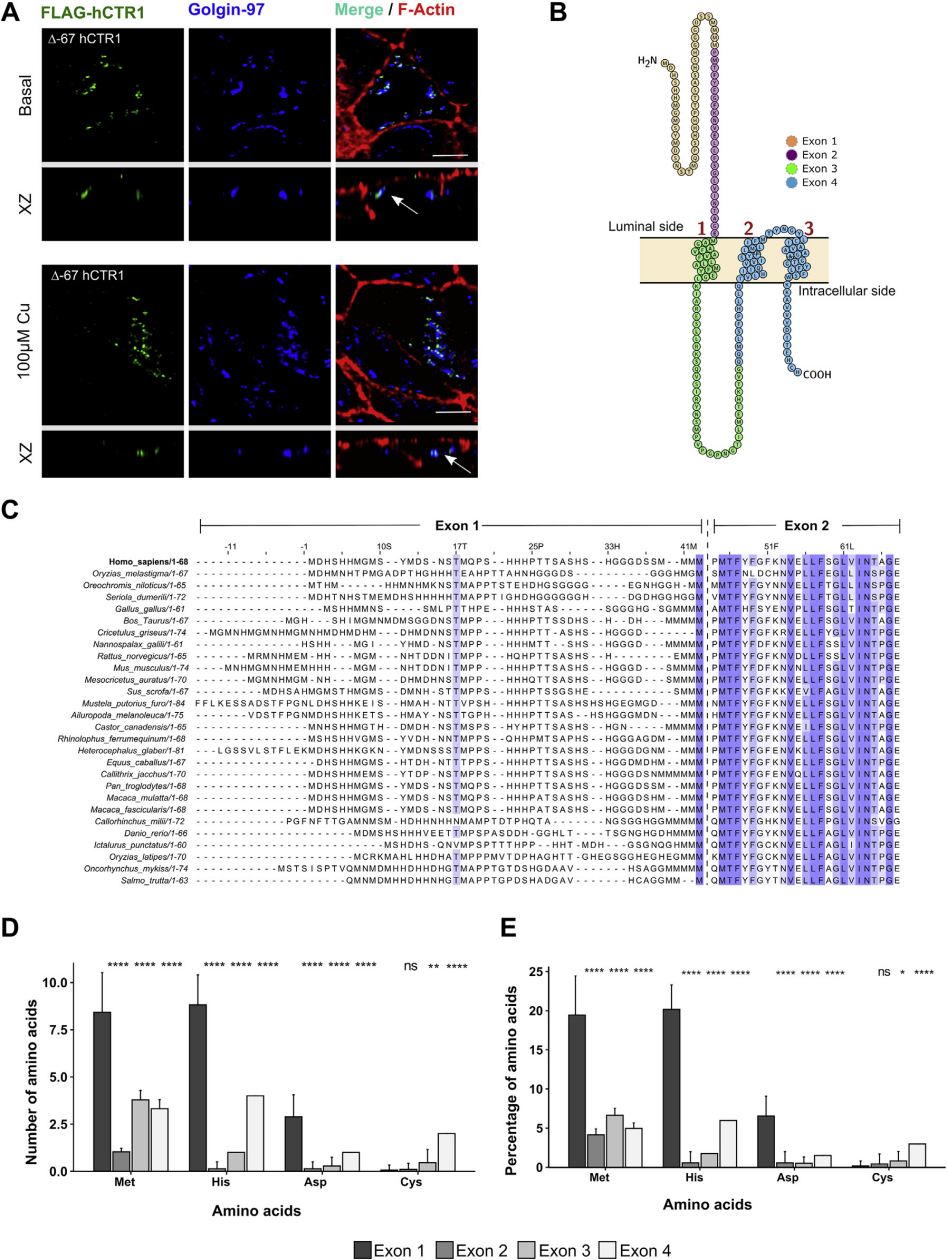
**His-Met rich N-term of hCTR1 is crucial for its plasma membrane localization.***A*, amino-terminal deleted Flag-Δ-67 hCTR1 (*green*) colocalizes with TGN-marker Golgin-97 (*blue*), both in basal (*upper panel*) and 100 μM copper treated condition (*lower panel*). [scale bar- 5 μm]. *B*, amino acids of hCTR1 monomer color coded based on the respective exons encoding them. Representative image was made in protter. *C*, sequence alignment of the first two exons of chordata CTR1s shows higher variability in the first exon as compared with the second one. Conserved residues are marked by *blue color*. Intensity of *blue color* is proportional to the conservation status of a residue. Bar plot (mean ± SD) representing the number (*D*) and percentage (*E*) of His, Met, Asp and Cys residues, color coded by the four exons of CTR1 from 28 chordate species. ∗*p* < 0.05, ∗∗*p* < 0.01, ∗∗∗∗*p* < 0.0001; ns, not significant (nonparametric Mann–Whitney U test/Wilcoxon rank-sum test). hCTR1, human copper transporter-1.

The N-terminus of hCTR1 forms the extracellular domain that is exposed to copper from the basolateral side of polarized epithelial cells. Given that, the cytosolic C-term should first sense the apically incorporated copper, fails to induce endocytosis of the protein. So we hypothesized that it is the N-term (1–67 amino acids) but not the C-term that primarily senses the extracellular copper and induces subsequent early physiological responses. It is possible that the changes in the N-terminus conformation upon copper binding are relayed on to the C-terminus that eventually leads to endocytosis of the protein. To test that, we deleted the N-terminus (1–67) and determined intracellular localization of the truncated protein in basal and copper-treated conditions. Interestingly, we found that Δ67-hCTR1 though expresses well and folds properly to exit ER, fails to localize at the basolateral membrane. Instead, Δ67-hCTR1 localizes on the TGN at basal or high-copper conditions [Fig. 2*A* (top and bottom panel)]. We can conclude that the N-term is crucial for plasma membrane localization of hCTR1 that is either regulated by copper binding to the N-term or by rendering the protein in TGN-exit favorable conformation. Deleting the entire N-term did not provide us a detailed view of its role in copper sensing and copper uptake. However, previous study by Eisses *et al.* (42) showed that this same truncated form can uptake copper at a very low level in sf9 cells.

### The distal aminoterminal domain exhibits higher phylogenetic variability as compared with the proximal part

A comprehensive sequence alignment and homology analysis of 219 CTR1s across species reveals that the extracellular amino-terminal domain is not evolutionarily conserved (Fig. S2). In contrast, the hCTR1 TM domains and the cytosolic tail exhibit higher phylogenetic similarity and contain conserved motifs such as the ^150^MXXXM^154^ in TM2 and ^188^HCH^190^ in the cytosolic tail (26, 43). Chordate CTR1 is mostly composed of four exons, and the domain architecture encoded by the four exons is illustrated and color-coded in Figure 2*B*. For *hCTR1*, the first two exons code for the extracellular aminoterminal domain. The third one codes for the first TM (TM1) domain and part of the cytosolic loop flanked by TM1-TM2. The fourth exon encodes for the central pore-forming region of the protein necessary for copper transport and the cytosolic C-terminal domain (44).

A relatively high conserved amino-acid sequence of the ‘‘channel-pore forming exon” coded by exons 3 and 4 (illustrated in Fig. S3*A*) suggests that the mechanism of copper import is conserved across the species. The higher variability in the first exon could be attributed to the differences in copper sensing and acquiring mechanisms in different species growing in different environments. Interestingly, sequence conservation also varies within the N-term. The distal part (away from the TM1) of the N-term (amino acid 1–42) is less conserved, and the proximal half (toward TM1, from amino acid 43–67) is relatively more conserved among various species (Fig. 2*C*). Exon1, despite having higher variability, exhibits bias toward certain residues, namely histidine, methionine, and aspartic acid, in terms of total abundance as well as percentage (Figs. 2, *D* and *E* and S3*B*). In line with previous studies (26), we hypothesize that the higher prevalence of a few amino acids is functionally relevant and participates in copper coordination and transport.

Interestingly, the His and Met residues are found in clusters rather than being dispersed along the primary sequence.

Upon comparison of the N-terminal amino acid sequence in 28 chordate species, we found that the clusters share a distinct homology, although they are not present in identical regions. Four discrete and unique clusters with varying homology were identified (Fig. S3*C*).

### N-terminal His-Met clusters and aspartate residues are putative Cu(II) and Cu(I) coordination residues

The N-terminal domain of hCTR1 has been predicted to be unstructured (14, 45, 46). Despite multiple trials using various affinity tags, we failed to purify the full-length N-term of hCTR1 for copper-binding assays. The sequence comparisons presented above suggest that the shorter synthetic peptide (^1^M-^14^S) construct that has been previously reported will provide an incomplete understanding as it lacks the key His-Met motifs (^31^HSH^33^ and ^40^MMMMPM^45^) of the proximal N-term (25). Consequently, we explored the molecular mechanism of the copper binding at the N-terminal domain using a combination of classical molecular dynamics, enhanced sampling, and quantum mechanical approaches to probe the free-energy surface of complex processes. In the molecular dynamics simulations, the Cu(II)/Cu(I) ion is represented as a virtual site model that allows us to consider the coordination geometry of the metal ion without electronic structure calculations (47, 48). Enhanced sampling methods, in conjunction with experiments, have been shown to be well suited to provide critical insights into the binding and unbinding process by virtue of predicting binding constants and molecular mechanisms (49). In the first step, we independently simulated copper ion in both oxidation states, Cu(II)/Cu(I), in conjunction with the N-terminal domain (system setup is shown in Fig. S4*A*). From the simulations, we observe transient interactions of Cu(II)/Cu(I) with different sites on the N-terminal domain. The high structural flexibility and relative abundance of histidine and methionine in spatially nearby regions required a more comprehensive sampling of Cu(I) and Cu(II) association in hCTR1. To refine the different interaction sites, well-tempered metadynamics simulations were used to calculate the binding free energy of the Cu(II)/Cu(I) ion along a pathway described by two collective variables (described in Fig. S4, *B* and *C*). The collective variables are chosen independently for the Cu(I/II) simulations and are centered at the Met/His rich clusters sites (based on the distance and angle from these sites). Details of the simulation are presented in Fig. S4*D*.

**Figure 3:**
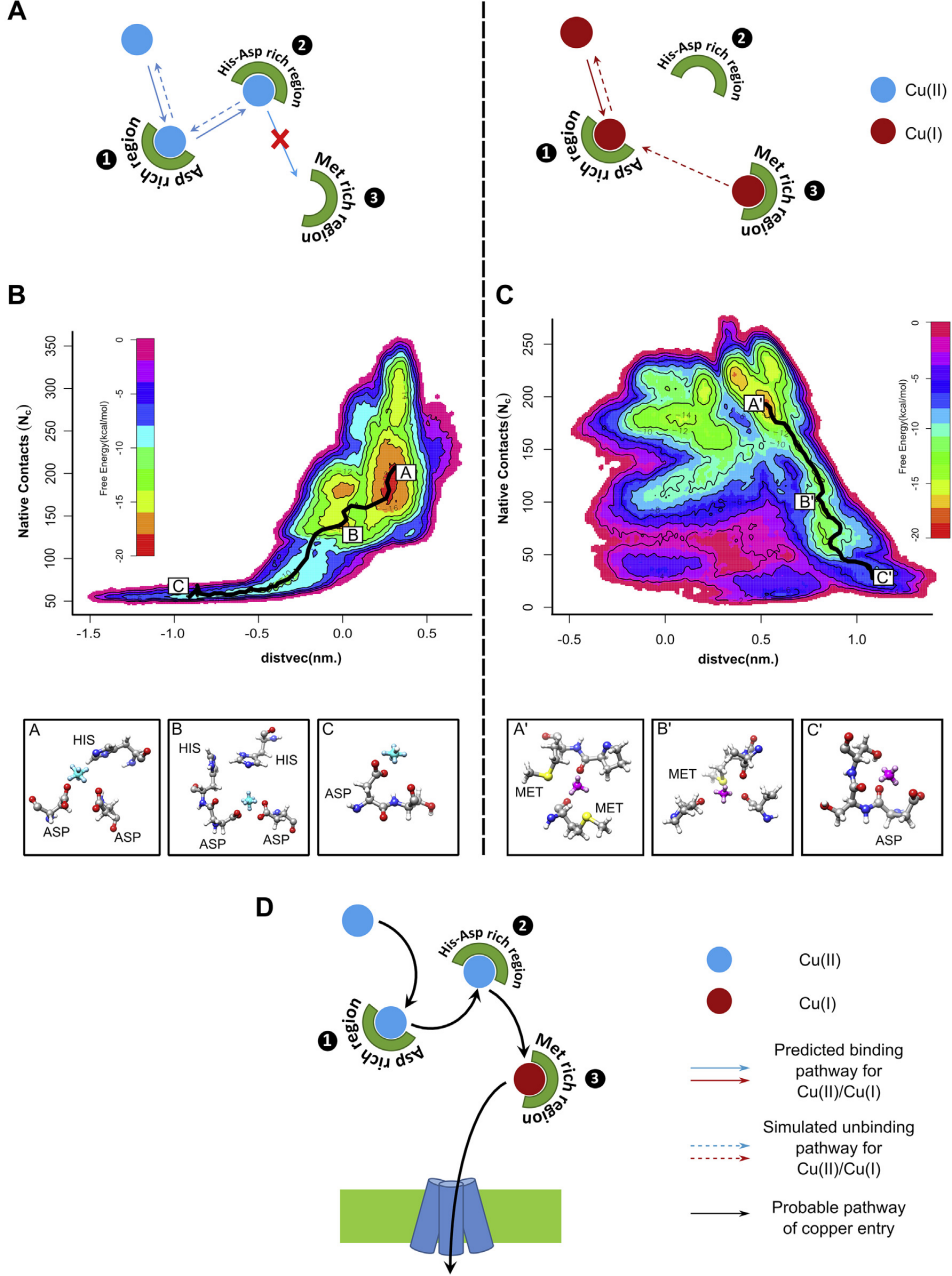
**N-term aspartic acids and histidines coordinate Cu(II), whereas methionines are primarily responsible for binding to Cu(I).***A*, schematic of the molecular mechanism as probed through our metadynamics simulations for both Cu(II) (*left panel*) and Cu(I) (*right panel*).Free energy surface of Cu(II) (*B*) and Cu(I) (*C*) binding to trimeric hCTR1 N-term (67 amino acids) against two collective variables, Native contacts (N_C_) and distvec (nm). The structures along the path of dissociation are shown below the free energy diagram (*A*, *B*, and *C* indicate the different Cu(II) coordination complexes, whereas A', B', and C' indicate the different Cu(I) complexes). Cu(II) octahedral model is shown in cyan, and the Cu(I) tetrahedral model is shown in *magenta*. *D*, schematic of the proposed pathway of copper shuttling through the N-term. hCTR1, human copper transporter-1.

A schematic diagram elucidating the molecular mechanism of Cu(II) binding to the N-term as probed through our metadynamics simulations is shown in Figure 3*A*. The converged free-energy profiles consist of a series of minima corresponding to specific sites for ion–protein interactions along the binding/unbinding pathway. The potential binding pathways of the Cu(II) ion were computed using the nudged elastic band method (50), and the main clustered structures were identified (Fig. 3, *B* and *C*). Our results indicate that the binding (or equivalently, the unbinding) of the Cu(II) octahedral virtual site model occurs at histidine-rich sites (Fig. 3*B*). The bioinorganic complexes observed contain coordination bonds with imidazole nitrogen atoms from a nearby histidine residue and two carboxylate groups from two proximal aspartate residues. The coordination sphere of the octahedral Cu(II) was further complemented by the solvent molecules. The subsequent unbinding of copper gives rise to a highly hydrated Cu(II) state commonly bound to a single carboxylate group only. For Cu(II), log K has been calculated to be 13.66 from the most probable binding pathway on the computed and converged free-energy surface. Considering K_d_ = 1/K, the dissociation constant, when converted to free energy at 300K temperature, yields a value of 18.76 kcal/mol. Our data matches quite well with that of Stefaniak *et al.*, (25) who have provided a log K value of 13.2 ± 0.3, for Cu(II) binding to the model peptide hCTR1_1–14_ through NTA competition assay; on the contrary, a much lower log K value for Cu(II)- hCTR1_1–14_ as well as for Cu(II)- hCTR1_1–55_ has also been reported in the literature, although they did not explicitly take into account the interference of buffer and other solution components (51, 52).

In the next step, we performed a similar metadynamics simulation for the tetrahedral Cu(I) virtual site model to analyze the binding of the Cu(I) ion at the N-terminal domain. Upon inspection of clustered structures on the free-energy surface along the minimum free energy path (Fig. 3*C*), we observe that Cu(I) binding occurs in exclusively methionine-rich regions, in stark contrast to the computationally predicted complexes of Cu(II). The bound state contains up to two methionine residues coordinated *via* their sulfur atoms. Upon unbinding, the complexes transition into relatively oxygen donor-rich complexes before becoming fully hydrated and thus fully unbound. In the case of Cu(I), our calculated log K comes around 13.17, which at 300 K corresponds to a free-energy value of 18.084 kcal/mol. However, our data are not in very good agreement with studies carried out by Du *et al.* and Yang *et al.*, (52, 53) where they observe log K values of 14.92 and 14.7 for Cu(I) for hCTR1_1–55_ and hCTR1_1–46_, respectively, through competition reactions with bicinchoninic acid (BCA). The difference in the values of dissociation (or binding) constants could arise from the difference in the reference considered. The free energy from our simulations is calculated considering a completely solvated structure of Cu(I) as the fully unbound state, whereas under experimental conditions, Cu(I) always remains in complex with some ligands and never as just a fully hydrated ion.

We substantiated our findings on the less well-established Cu(I) site using quantum mechanical calculations. To validate the Cu (I) complexes observed in the simulations with the virtual site Cu(I) model, we performed first-principles density functional theory (DFT) calculations on representative structures. We considered representative clusters from the simulations, and the stability of the corresponding isolated Cu(I) coordinated residues was tested by short simulations. Five such structures were then considered for the gas-phase DFT optimizations (no surrounding water molecules were considered). For the DFT calculations, the Cu atom was initially considered with +1 charge. The initial and optimized structures are shown in Fig. S5. The convergence of all five structures was observed, and the deviation from the initial positions is low. It was observed in the optimized structures that the clusters were stable and the molecules interact with Cu through O as well as S. Spin-polarized calculations showed that the converged structures are nonmagnetic. Overall, it is evident that the virtual site Cu(I) model is well able to represent its interactions with the peptide.

Our results suggest that both Cu(II) and Cu(I) can bind to the N-terminal domain with very disparate binding modes. This is also the first time we show that there is a possible role of aspartate residues found abundantly on hCTR1 N-term, primarily in the Cu(II)-binding process. This hypothesis is further tested by experiments in the following sections. A probable pathway of Cu(II) and Cu(I) shuttling through the N-term before it enters the TM pore is shown in Figure 3*D*.

### N-terminal methionine cluster and aspartate facilitate copper uptake

**Figure 4:**
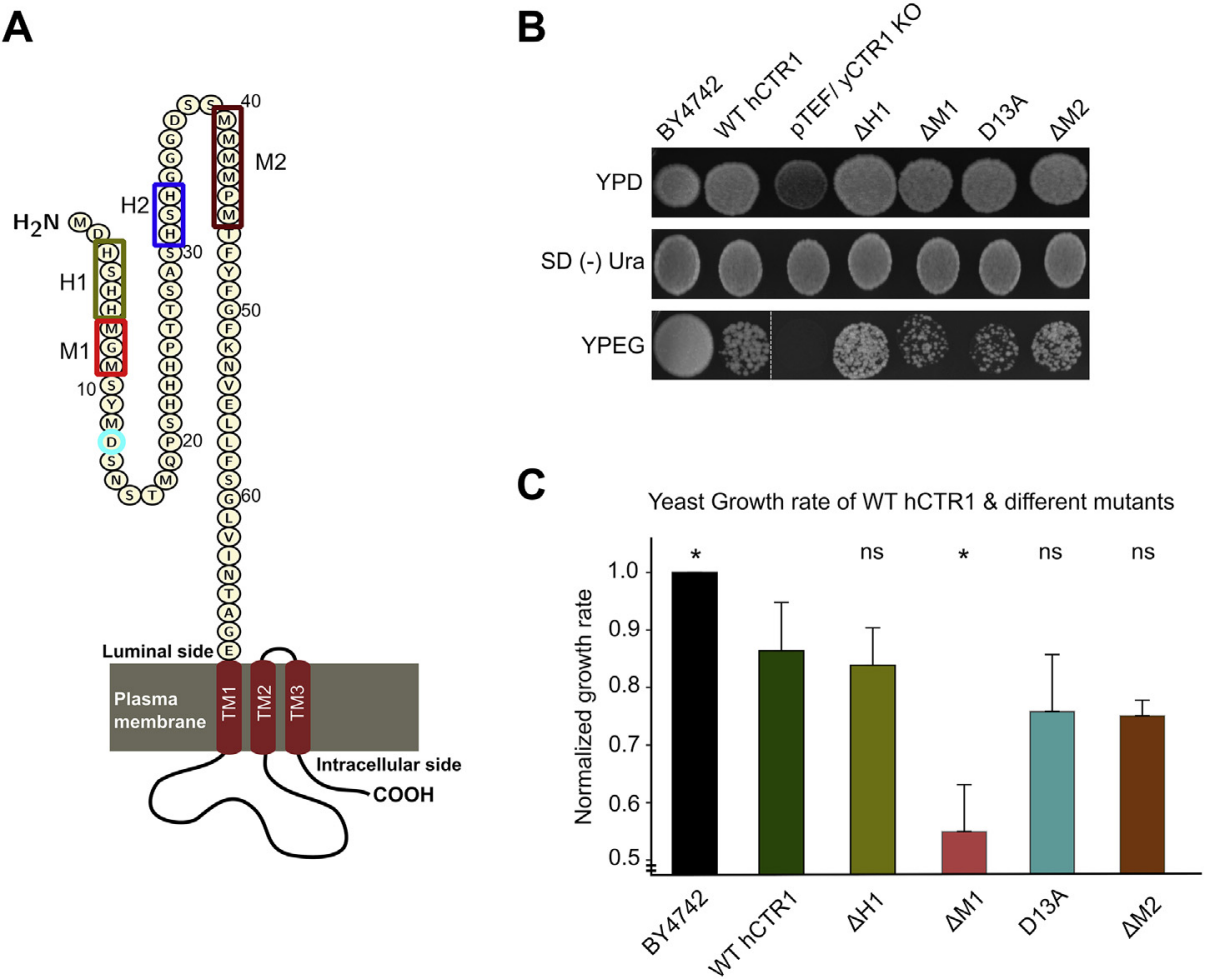
**hCTR1 N-term methionine and aspartic acid residues are critical for copper import.***A*, illustration of the 67 amino acid long hCTR1 N-term; *color-coded rectangles* and *circles* indicate the position of different histidines, methionines clusters, and aspartic acid mutations used in yeast complementation assays. *B*, in the YPD plate, all the constructs show appreciable growth. We selected the mutants in SD (-) Ura plate following transformation. In the YPEG plate, the empty vector (pTEF) containing strain (Δ*yCTR1BY4742*) fails to grow but WT-hCTR1 is able to successfully complement the ΔyCTR1 growth defect. *C*, normalized growth rate of WT and mutant hCTR1s (mean ± SD) shows significantly reduced growth in ΔM1 (ΔM7-M9) mutant, followed by D13A and ΔM2 (ΔM40-M45). ΔH1 (ΔH3-H6) shows almost similar growth rate as that of WT. Statistical significance is denoted by ∗*p* < 0.05; ns, not significant (nonparametric Mann–Whitney U test/Wilcoxon rank-sum test w.r.t WT hCTR1). Sample size for each set, n = 4. BY4742 is the wild-type yeast strain, containing endogenous yCTR1 and in the remaining cases wild-type and mutant hCTR1s are separately transformed into the yCTR1 deleted strain; “∗” signifies *p*-value less than 0.05. hCTR1, human copper transporter-1.

To understand the role of copper binding to the amino-terminal residues (predicted in the previous section) in its subsequent uptake by hCTR1, we utilized yeast complementation assay. We expressed the WT hCTR1 and multiple hCTR1 N-terminal mutants (illustrated in Fig. 4*A*) in Δ*yCTR1* (*BY4742 Saccharomyces cerevisiae* strain lacking endogenous *yCTR1*). Yeast growth rate phenotype was used as an indicator of copper uptake in restrictive media (54). We measured yeast growth using nonfermentable carbon sources (ethanol and glycerol) containing media as it will allow the growth of only those colonies that are able to import copper through ectopically expressed hCTR1 (WT or mutant hCTR1). Subsequently, the imported copper will activate the cytochrome c oxidase of mitochondria for ATP production. Thus, cell growth in this YPEG (Yeast extract, Peptone, Ethanol, and Glycerol) media is proportional to the copper import property of the transformed *hCTR1* constructs (details provided in Experimental procedures section). We measured yeast growth both qualitatively and quantitatively (in the plate and in liquid culture media, respectively). Empty pTEF vector transformed strain showed no growth in YPEG plate, and WT hCTR1 (cloned in pTEF) recovered growth, indicates that human CTR1 is able to complement yCTR1 (Fig. 4*B*). There are three separate clusters of histidines, ^3^HSHH^6^, H^22–24^, ^31^HSH^33,^ that are postulated to bind Cu (II). In solid media culture using colony counting, we found that ΔH^3^-H^6^ mutants exhibited similar growth patterns to the WT hCTR1. Lower colony counts in ΔM^7^-M^9^ and D13A mutants indicate reduced growth in comparison to WT (Fig. 4*B*). Growth kinetics was measured during the log phase in media culture that is otherwise not possible in end-point colony counting in plate cultures. Replicating the data from colony counts, ΔM^7^-M^9^ and D13A, showed a reduced growth rate, *i.e.*, 36% and ∼15%, respectively, than the WT indicating reduced copper import. ΔH^3^-H^6^ expressing yeast showed no alteration in the growth rate compared with the one expressing WT-hCTR1, which signifies that copper uptake property of this mutant is unaltered. ΔM^40^-M^45^ mutant showed ∼15% reduced growth as compared with the WT (Fig. 4*C*). To summarize, the methionine clusters, especially the first one, constitute the key motif that participates in copper uptake.

### ^7^MGM^9^ and D^13^ are indispensable for copper-induced hCTR1 endocytosis

**Figure 5:**
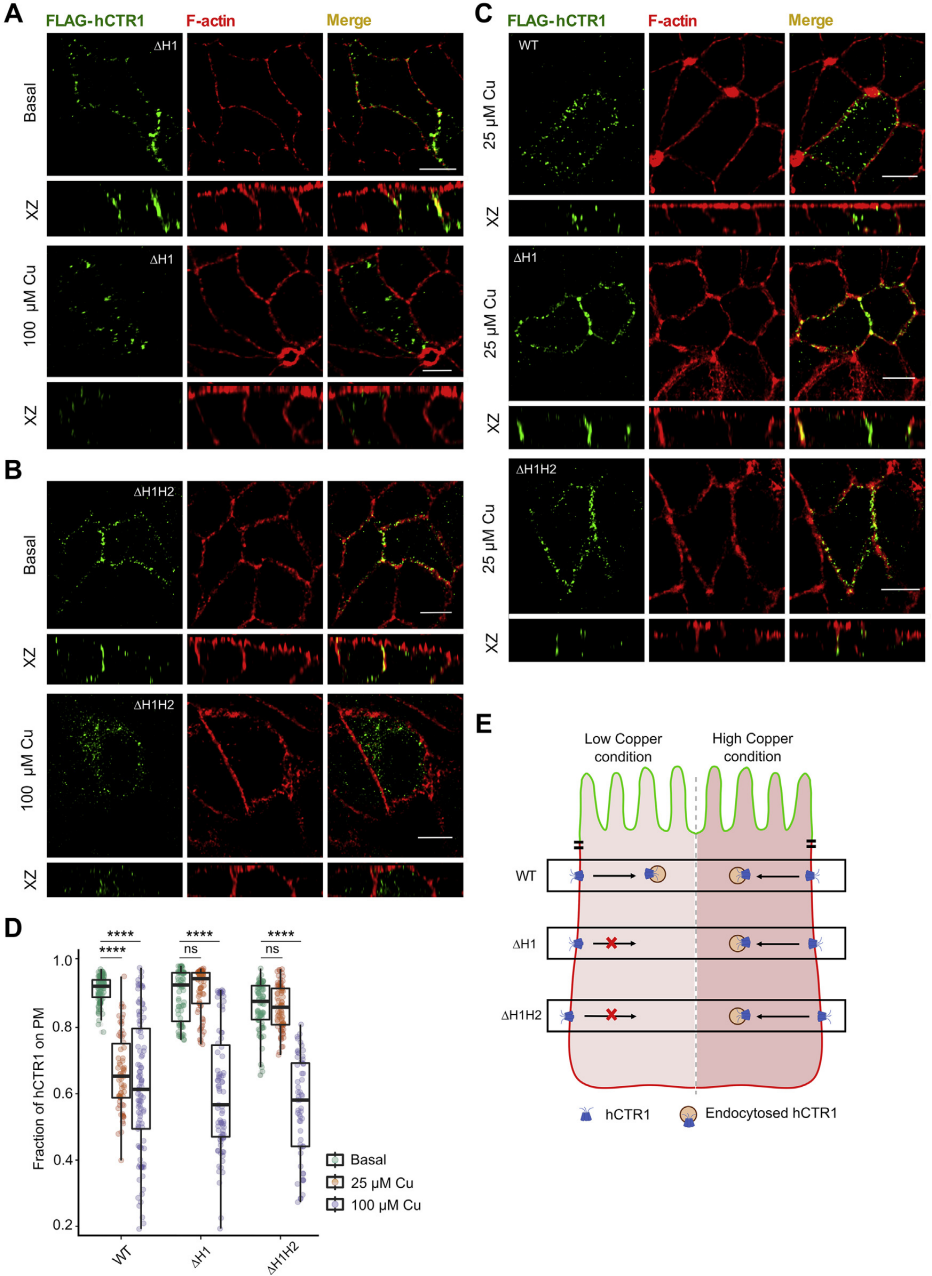
**N-term histidines are crucial for hCTR1 endocytosis under copper-limiting conditions.***A*, ΔH1 Flag-hCTR1 (*green*) localizes at the basolateral membrane at basal copper (*upper panel*) and endocytoses upon 100 μM copper treatment (*lower panel*). *B*, ΔH1H2 (combined deletion of ΔH3-H6 and ΔH31-H33) under basal (no copper treatment) condition resides on the PM (*upper panel*). During 100 μM copper treatment, this mutant shows endocytosis (*lower panel*). *C*, WT hCTR1 (*upper panel*) endocytoses under low (25 μM) copper treatment, whereas, under similar treatment conditions, both ΔH1 (*middle panel*) and ΔH1H2 (*lower panel*) mutants fail to do so. *D*, fraction of hCTR1 colocalization with membrane marker F-actin, demonstrated by box plot with jitter points under basal (*green circles*), 25 μM copper (*orange circles*), and 100 μM copper treated (*purple circles*) conditions. The box represents the 25 to 75th percentiles, and the median in the middle. The whiskers show the data points within the range of 1.5 × interquartile range (IQR) from the first and third quartile. ∗∗∗∗*p* < 0.0001; ns, not significant (nonparametric Mann–Whitney U test/Wilcoxon rank-sum test). Sample size (n) for WT (basal: 100, 25 μM Cu: 66, 100 μM Cu: 101), ΔH3-H6 (basal: 78, 25 μM Cu: 70, 100 μM Cu: 83), ΔH1H2 (basal: 76, 25 μM Cu: 77, 100 μM Cu: 56). [In all the conditions, cells are polarized MDCK-II, XZ section shows the orthogonal sections of all the stacks, *green*- FLAG-hCTR1 and *red*- F-actin; copper treatment- 25 μM and 100 μM separately on the basolateral chamber of the transwell]. [scale bar: 5 μm for all the images in (*A–D*)]. *E*, schematic summarizing the phenotypes of the WT and the His-mutants under low- and high-copper conditions. hCTR1, human copper transporter-1; MDCK, Madin Darby Canine Kidney.

As a self-regulatory mechanism to limit intracellular copper concentration, hCTR1 endocytoses from the plasma membrane upon copper treatment (16). We investigated if copper binding to the N-term, copper import, and endocytosis are linked and interdependent to maintain the proper functioning of the protein. To study that, at the outset, we deleted the first His motif, ^3^HSHH^6^ (ΔH1). In agreement with unaltered copper uptake observed using yeast complementation assay, the mutant localizes on the basolateral membrane in basal condition and endocytoses in elevated copper (100 μM) comparable with the wild-type (WT) protein (Fig. 5*A*). Interestingly, even upon deletion of both the His cluster ^31^HSH^33^and ^3^HSHH^6^ (double mutant, ΔH1H2), we did not notice any deviation in phenotype in basal or elevated copper compared with the wt-CTR1 and ΔH^3^-H^6^ (Fig. 5*B*). Based on our computational findings, we hypothesize that the His motifs sequester Cu(II) from the environment and increase its local availability for further reduction and uptake. So the role of His motifs would be more apparent in copper-limiting conditions. Upon treating the cells with lower copper (25 μM), both the abovementioned single His motif mutant and the double His motif mutant failed to endocytose though WT protein endocytoses under similar low copper concentrations (Fig. 5*C*). These results point to an essential role of the N-terminal histidine motifs in acquiring copper under physiological low copper concentrations and thereby promoting hCTR1 endocytosis. Presence of high copper, *i.e.*, 100 μM, “undermines” the importance of histidine motifs as the Met clusters can as well bind and sequester Cu(II), though with a lower affinity. Quantitation of endocytosis of the WT and the two His mutants under different copper concentrations has been illustrated in Figure 5*D*. A schematic summarizing the phenotypes of the WT and the mutants under low and high copper conditions has been elucidated in Figure 5*E*.

**Figure 6:**
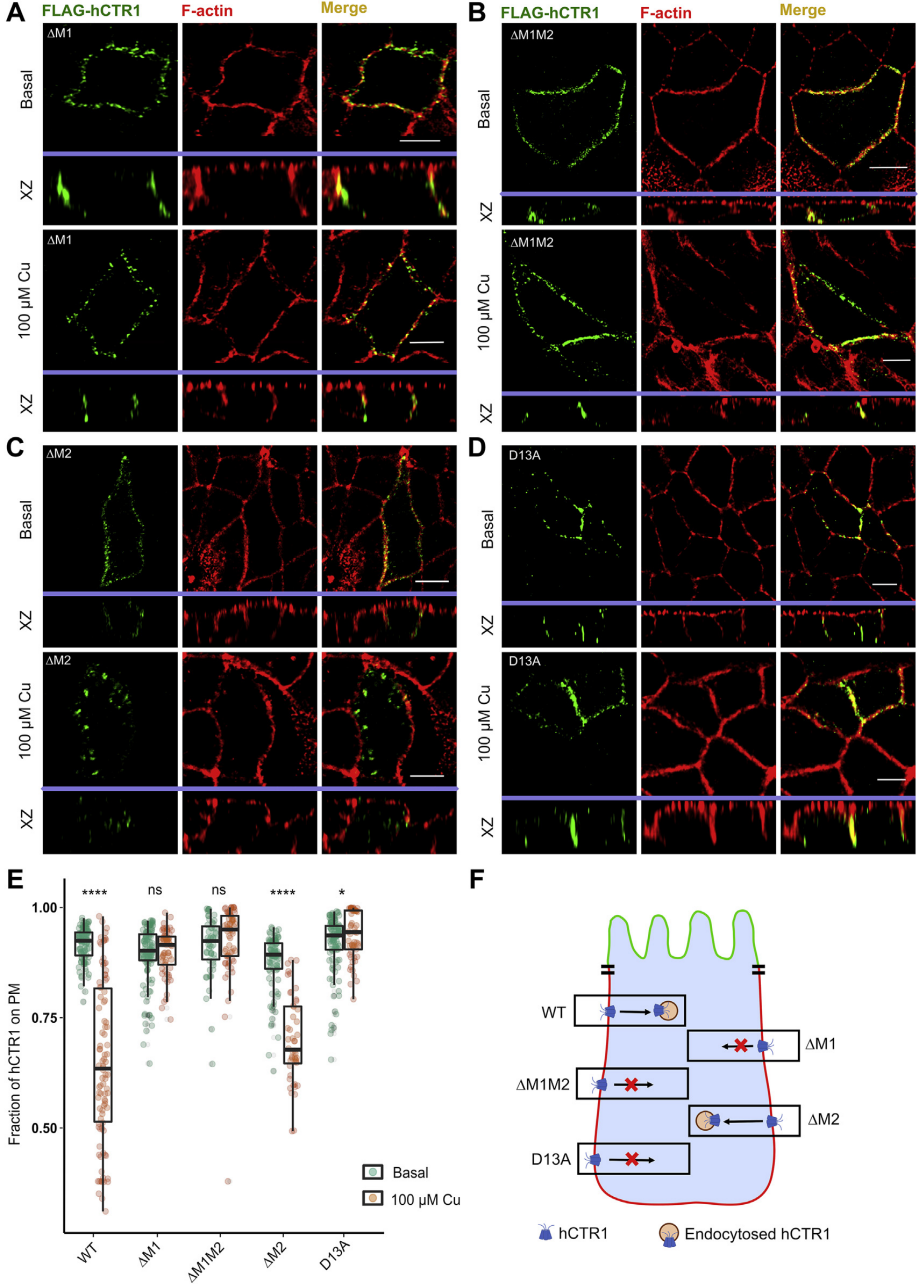
**hCtr1 N-term methionines and aspartic acid regulates endocytosis of hCTR1.***A*, ΔM1 Flag-hCTR1) localizes at the basolateral membrane at basal copper (*upper panel*) and fails to endocytose under treatment with 100 μM copper (*lower panel*). *B*, ΔM1M2 Flag-hCTR1 (combined deletion of ΔM7-M9 and ΔM40-M45) localizes at the basolateral membrane at basal copper (*upper panel*), it fails to endocytose under treatment with 100 μM copper (*lower panel*). *C*, ΔM2 Flag-hCTR1 (ΔM40-M45) localizes at the basolateral membrane at basal copper (*upper panel*) and endocytoses upon 100 μM copper treatment (*lower panel*). *D*, D13A localizes at the basolateral membrane at basal copper (*upper panel*) and fails to endocytose upon 100 μM copper treatment (*lower panel*). *E*, fraction of hCTR1 colocalization with membrane marker F-actin, demonstrated by box plot with jitter points under both basal (*green circles*) and 100 μM copper treated (*orange circles*) conditions. The box represents the 25 to 75th percentiles, and the median in the middle. The whiskers show the data points within the range of 1.5 × interquartile range (IQR) from the first and third quartile. ∗*p* < 0.05, ∗∗∗∗*p* < 0.0001; ns, not significant (nonparametric Mann–Whitney U test/Wilcoxon rank-sum test). Sample size (n) for WT (basal: 100, Cu: 101), ΔM1 (basal: 109, Cu: 61), ΔM1M2 (basal: 51, Cu: 65), ΔM2 (basal: 93, Cu: 48), D13A (basal: 106, Cu: 57). [In all the conditions, cells are polarized MDCK-II, XZ section shows the orthogonal sections of all the stacks, *green*: FLAG-hCTR1 and *red*: F-actin; 100 μM Cu treatment on the basolateral chamber of the transwell]. [scale bar: 5 μm for all the images in (*A–E*)]. *F*, schematic summarizing the phenotypes of the WT and the different Met and Asp-mutants under high-copper conditions. hCTR1, human copper transporter-1; MDCK, Madin Darby Canine Kidney.

hCTR1 contains two N-terminal methionine clusters (M1; ^7^MGM^9^) and (M2; ^40^MMMMPM^45^). To determine their role in the localization and copper-induced endocytosis of the protein, we deleted M1 and M2 individually (ΔM1 and ΔM2) and both the clusters together (ΔM1M2). In basal copper conditions, all these three mutants localized on the plasma membrane. Interestingly, upon copper treatment, ΔM1 failed to endocytose from the basolateral membrane (Fig. 6*A*). A similar nonendocytosing phenotype was also observed in the double mutant ΔM1M2 (Fig. 6*B*). Interestingly, upon deleting M2 only, the protein endocytosed upon copper treatment, similar to WT-hCTR1 (Fig. 6*C*).

Our simulation studies, for the first time, also indicated the importance of aspartates explicitly for Cu(II) coordination. The N-term contains three aspartate residues, namely- Asp^2^, Asp^13^, and Asp^37^. Asp^2^ is considered to be part of the well-known ATCUN- cluster (comprising the first three amino acids, Met-Asp-His), which is thought to be indispensable for Cu(II) binding (55). On substituting Asp^13^ by Ala (D13A), we found that the mutant failed to endocytose under elevated copper conditions (Fig. 6*D*). Quantitation of endocytosis of the Met and the Asp mutants under basal and high copper treatment conditions has been illustrated in Figure 6*E*. A schematic elucidating the phenotypes of the WT and the above-mentioned mutants under copper excess conditions has been illustrated in Figure 6*F*.

### Cu(I) and not Cu(II) triggers hCTR1 endocytosis

We know that intracellular copper remains bound to proteins in its reduced form (+1), whereas in the extracellular milieu, copper mostly exists in its higher oxidation state (+2) (56, 57). Serum albumin has been demonstrated to bind Cu(II) and deliver it directly to the N-term of hCTR1 (56). We speculated a role of the hCTR1 N-term, in regulating the reduction of Cu(II) to Cu(I), along with external reducing agents such as ascorbate or STEAP (58, 59). Ascorbate has been traditionally used to reduce Cu(II) to Cu(I). However, prolonged and inadvertent exposure to oxidizing agents during an experimental procedure can reoxidize Cu(I) to Cu(II) even in the presence of ascorbate. Additionally, uncoordinated Cu(I) exhibits very low solubility in aqueous media.

**Figure 7:**
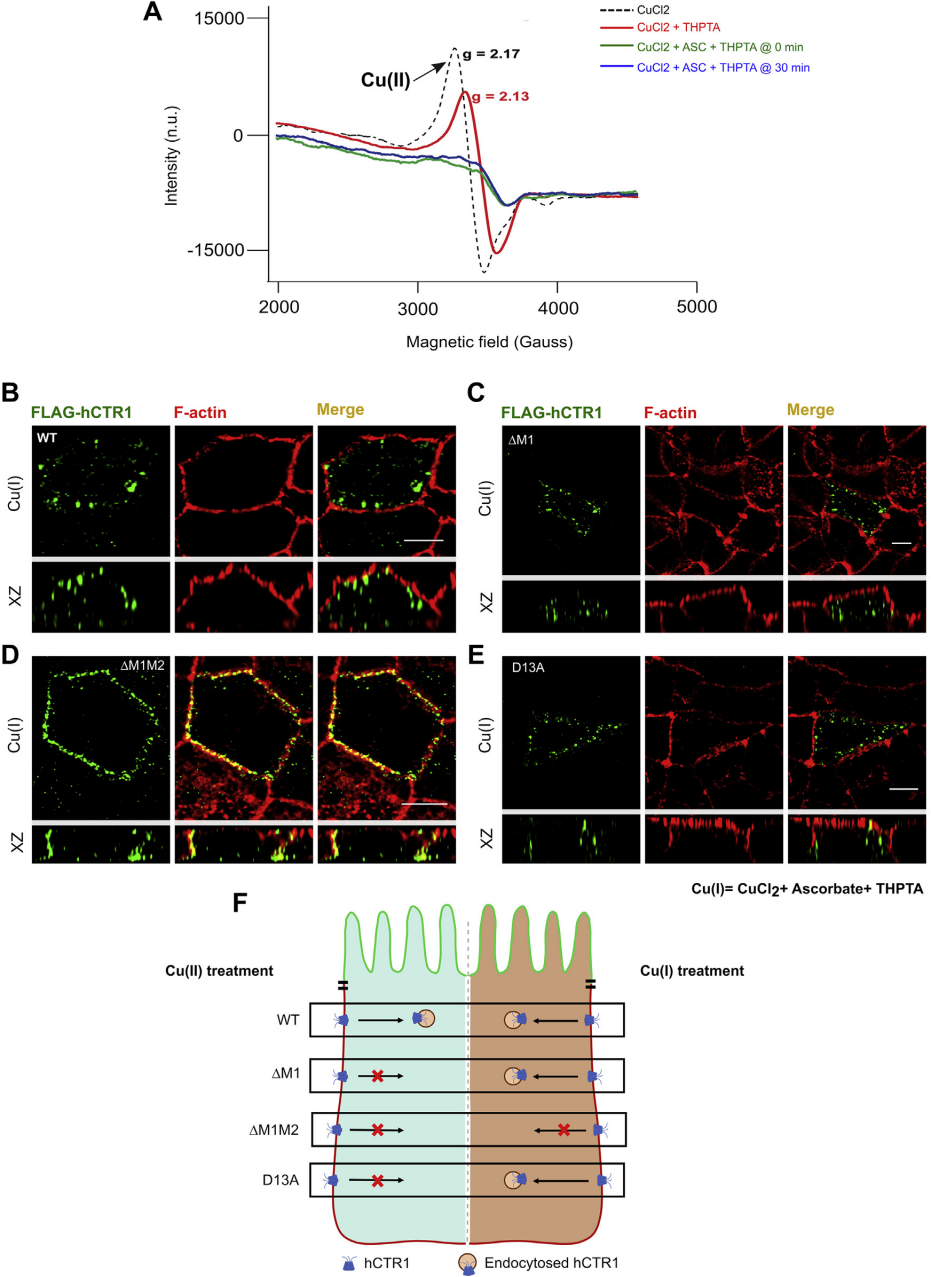
**Cu(I) treatment triggers endocytosis of the otherwise nonendocytosing hCTR1 amino-terminal mutants.***A*, EPR data shows the absence of Cu(II) and presence of only Cu(I) under ascorbate +THPTA + CuCl_2_ (2:2:1) treatment. The peak for Cu(II) does not reappear even after 30 min, which is the entire duration of our following *in-vivo* experiments. *B*, WT hCTR1 (*green*) endocytoses when treated with ascorbate +THPTA + CuCl_2_. *C*, nonendocytic ΔM1 mutant endocytoses when treated with Cu(I). *D*, ΔM1M2 Flag-hCTR1 retains its nonendocytic phenotype even under Cu(I) treatment (as marked by its colocalization with phalloidin staining F-Actin (*red*). *E*, non-endocytic D13A Flag-hCTR1 mutant endocytoses when treated with Cu(I). [In all the conditions, cells are polarized MDCK-II, XZ section shows the orthogonal sections of all the stacks, *green*: FLAG-hCTR1 and *red*: F-actin; (200 μM ascorbate +200 μM THPTA +100 μM CuCl_2_) treatment on the basolateral chamber of the transwell, scale bar: 5 μm]. *F*, schematic summarizing the phenotypes of the WT and the different Met and Asp-mutants under Cu(II) and Cu(I) treatment conditions. EPR, electronic paramagnetic resonance; hCTR1, human copper transporter-1; MDCK, Madin Darby Canine Kidney.

Subsequently, to distinguish between residues involved in differential Cu(II) and Cu(I) binding, we decided to use THPTA (3 [tris(3-hydroxypropyltriazolylmethyl)amine), a water-soluble, highly effective ligand for copper-catalyzed azide-alkyne cycloadditions (CuAAC), along with CuCl_2_ and ascorbate to provide the cells with a direct source of Cu(I). THPTA not only helps in maintaining the Cu(I) oxidation state during the experimental tenure against aerial oxidation, it also enables the protection of biomolecules from oxidative damage by ROS species that might be generated during the reaction (60, 61). ^1^H NMR and ^13^C NMR are used to confirm the preparation of THPTA that is subsequently used in our experiments (Fig. S6, *A* and *B*). UV-Vis spectroscopy of CuCl_2_ along with THPTA (2.0 equivalence) and ascorbate (varying from 0.5 to 2.0 equivalence) showed that copper was reduced by the entire range of ascorbate used (Fig. S6*C*). Copper remains in its reduced form during the full duration of the treatment (30 min) with no trace of Cu(II), leading us to hypothesize that it is Cu(I) and not Cu(II) that actually promote the endocytosis of this copper transporter. Reoxidation of Cu(I) by H_2_O_2_ at the end of 30 min leads to the reappearance of the Cu(II) peak. (Fig. S6*D*). Cu(II) gives an absorption maximum at 800 nm as evident from the UV-Vis spectrum, which remains intact after the addition of 2 equivalence of THPTA, whereas further addition of 2 equivalence of ascorbate to the previous mixture causes the peak to vanish, indicating that Cu(I) has formed (Fig. S6*E*). Electronic Paramagnetic Resonance (EPR) data also confirmed our UV-Vis experiments (Fig. 7*A*). Taking into consideration that the so-formed Cu(I)-THPTA complex can donate Cu(I) to hCTR1 N-term, we treated the polarized MDCK-II cells expressing Flag-WT-hCTR1 with the mixture of ascorbate: THPTA: CuCl2 in 2:2:1 ratio in HBSS for 30 min. Conforming to our hypothesis, hCTR1 was found to endocytose in response to this Cu(I) treatment (Fig. 7*B*). We can summarize that, under physiological conditions, when provided by a source of Cu(II), the protein in combination with other external reducing agents reduces Cu(II) to Cu(I), and Cu(I) acts as the main inducer of hCTR1 endocytosis.

### Cu(I) treatment induces endocytosis of the otherwise nonendocytosing ΔM1 and D13A mutant

As determined in the last section, hCTR1 mutants ΔM1 and ΔM1M2 failed to endocytose under elevated copper conditions (Cu(II) treatment). Based on our MD prediction that methionine clusters bind to Cu(I), we hypothesize that these clusters might play a role in maintaining the Cu(I)-Cu(II) redox balance. We treated the methionine mutants ΔM1 and ΔM1M2 with the mixture of ascorbate: THPTA: copper in a 2:2:1 ratio, which acts as a direct source of Cu(I) (as mentioned earlier). Interestingly, ΔM1 is endocytosed (Fig. 7*C*), whereas the ΔM1M2-hCTR1 stays localized at the basolateral membrane (Fig. 7*D*). Upon endocytosis in response to Cu(I) treatment, ΔM1 behaves similar to the WT-hCTR1 and localizes in the CRE as determined by transferrin uptake assay (Fig. S6*F*). It can be inferred that the presence of at least one methionine cluster is required to bind to the Cu(I) species for its uptake and subsequent endocytosis of the protein.

D13A hCTR1, which remained localized on the PM under Cu(II) treatment, endocytosed in response to Cu(I) (Fig. 7*E*). This observation reinforces our finding that just like the Met cluster, the presence of at least one aspartate residue is required to promote hCTR1 endocytosis. We hypothesize that the aspartates might act as a transient binder for copper in both of its oxidation states during the reduction process and thereby facilitate shuttling of the ion from its histidine-bound +2 state to the adjacent methionine-bound +1 oxidation state. A schematic summarizing the contrasting phenotypes elucidated by the different Met and Asp-mutants under Cu(II) and Cu(I) treatment is shown in Figure 7*F*.

### His-Met-Asp motifs of the proximal and distal N-terminal domain functionally complement each other

**Figure 8:**
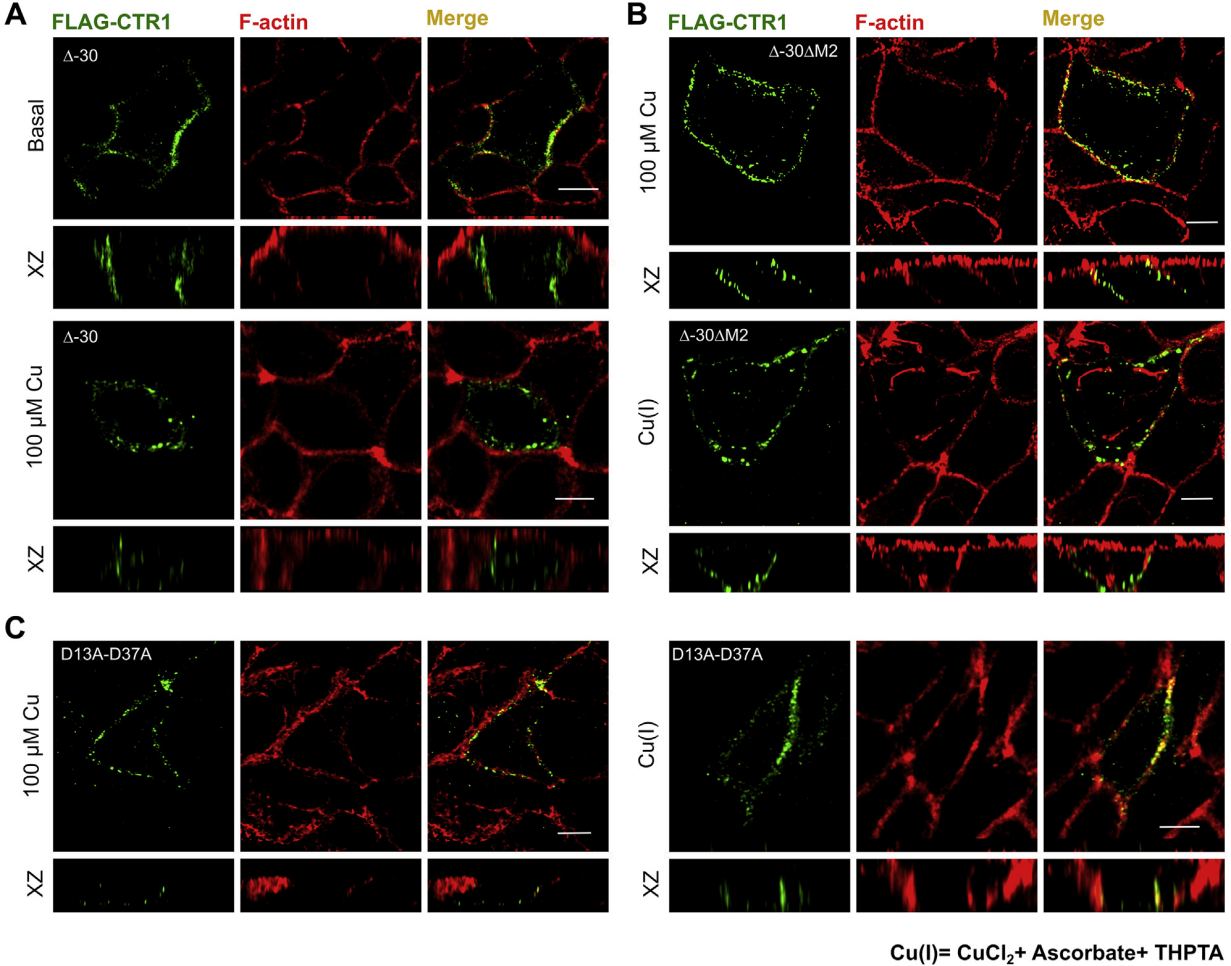
**Methionine clusters and Aspartate residues on the proximal and distal part of the hCTR1 aminoterminal exhibit complementarity.***A*, Δ-30-Flag-hCTR1 localizes at the basolateral membrane at basal copper (*upper panel*) and endocytoses in high copper (*lower panel*) (*B*) Δ30-ΔM2 Flag-hCTR1 mutant failed to endocytose when treated with both Cu(II) (*upper panel*) as well as with Cu(I) (*lower panel*), reminiscent of the phenotype exhibited by ΔM1M2 (see Figs. 6*B* and 7*D* respectively); (*C*) D13A-D37A Flag-hCTR1 mutant failed to endocytose when treated with both Cu(II) (*left panel*) as well as with Cu(I) (*right panel*). [In all the conditions, cells are polarized MDCK-II, XZ section shows the orthogonal sections of all the stacks, *green*: FLAG-CTR1 and *red*: F-actin; 100 μM Cu and (200 μM ascorbate +200 μM THPTA +100 μM CuCl_2_) treatment on the basolateral chamber of the transwell, scale bar: 5 μm]. hCTR1, human copper transporter-1; MDCK, Madin Darby Canine Kidney.

Analysis of the disposition of copper-coordinating amino acids on the N-term and subsequent experimental evidence point towards a possible functional complementarity of region 1 to 30 and 31 to 67 residues in the N-term of hCTR1. Few previous studies have shown that following copper-induced endocytosis, a cathepsin B mediated truncation of hCTR1 occurs at the amino terminus (37, 38). Maryon *et al.* (38) showed that hCTR1 undergoes O-linked glycosylation at Thr^27^, and blocking it facilitates a cleavage between the residues A^29^ and G^34^, giving rise to a truncated protein (lacking ∼first 29 amino acids) that endocytoses to Rab9 compartments in response to high copper. Though the truncated protein recycles back to the plasma membrane, it shows reduced copper-import property (42). To study the individual contribution of the proximal and the distal part of the N-term in copper transport leading to hCTR1 endocytosis, we generated the truncated construct, *i.e.*, Δ30-hCTR1. In basal copper, the deletion mutant localized to the basolateral membrane and endocytosed at elevated Cu(II) and Cu(I) conditions, mimicking the WT-hCTR1 phenotypes (Figs. 8*A* and S7*A*). Since in the Δ30-hCTR1 protein, M^7^-M^9^ (M1) is absent, we hypothesize that the reduction of copper, as well as Cu(I) binding, is facilitated by the second methionine stretch, ^40^MMMMPM^45^ (M2). Whether M2 can participate in the reduction or not probably depends on its extracellular solvent accessibility. The presence of the first 30 amino acids possibly causes a steric hindrance affecting the solvent accessibility of the proximal 31 to 69 amino acid stretch (closer to the transmembrane domain). Hence, ΔM1-hCTR1 lacking only M^7–9^ but retaining the rest of the amino acids of the aminoterminal distal half fails to facilitate reduction of Cu(II) and thereby fails to endocytose (Fig. 6*A*). However, in Δ30-hCTR1, the lack of the distal 1 to 29 residue stretch provides easy access of the proximal ^40^MMMMPM^45^ (M2) to copper in the culture media. In summary, M2 can functionally complement M1, though M1 is considered the principal mediator of endocytosis for the WT protein. In that line of thought, we generated Δ30hCTR1- ΔM2 that lacks both the Met clusters. Similar to the Met double mutant (ΔM1M2), Δ30hCTR1- ΔM2 failed to endocytose both under Cu(II) and Cu(I) conditions, supporting our hypothesis that indeed the two Met clusters complement each other and at least one Met cluster ensures near proper functioning of the protein (Fig. 8*B*).

In our previous experiment with 100 μM copper, Δ^3^HSHH^6^ showed copper-induced endocytosis (Fig. 5*A*). We investigated whether deleting the His cluster present on the proximal part of the N-term shows a similar phenotype. We observed that Δ30hCTR1- Δ^31^HSH^33^ (Δ30hCTR1- ΔH2) mutant exhibits normal localization on the plasma membrane and subsequent endocytosis upon 100 μM copper treatment similar to the WT protein and the Δ^3^HSHH^6^ mutant (Fig. S7*B*, *top and bottom panel*). Under low-copper conditions (25 μM), however, this mutant failed to endocytose completely and remained localized on the PM (Fig. S7*B*, *middle panel*).

As mentioned in the previous section, the single Asp mutant (D13A) failed to endocytose under Cu(II) treatment, whereas under direct Cu(I) application, it showed WT phenotype. Mutant 2D-2A (D13A-D37A), lacking both D13 and D37, failed to endocytose under both Cu(II) and Cu(I) treatment (Fig. 8*C*, left and right panels, respectively). This observation is in agreement with our previous hypothesis that the aspartates (D13 and D37) indeed play a crucial role in transiently binding and shuttling of Cu(I) to the nearest Met clusters (M1 and M2), respectively. Therefore, we can conclude that in the D13A mutant, D37 functionally complements D13 along with the adjacent methionine stretches, M^40^-M^45,^ in respectively transferring copper and binding Cu(I), thereby facilitating its uptake. The presence of at least one of either aspartates, D13 or D37, is warranted for the uptake of copper. Finally, we utilized immunoblotting to confirm the expected sizes and protein expression levels of all the mutants and the wt Flag-hCTR1 that is used in this study. Upon comparing with a housekeeping protein GAPDH, we observed that all the mutants express well compared with the wt-Flag hCTR1, and the truncation mutants are of sizes that we expect. (Fig. S7*C*).

**Figure 9:**
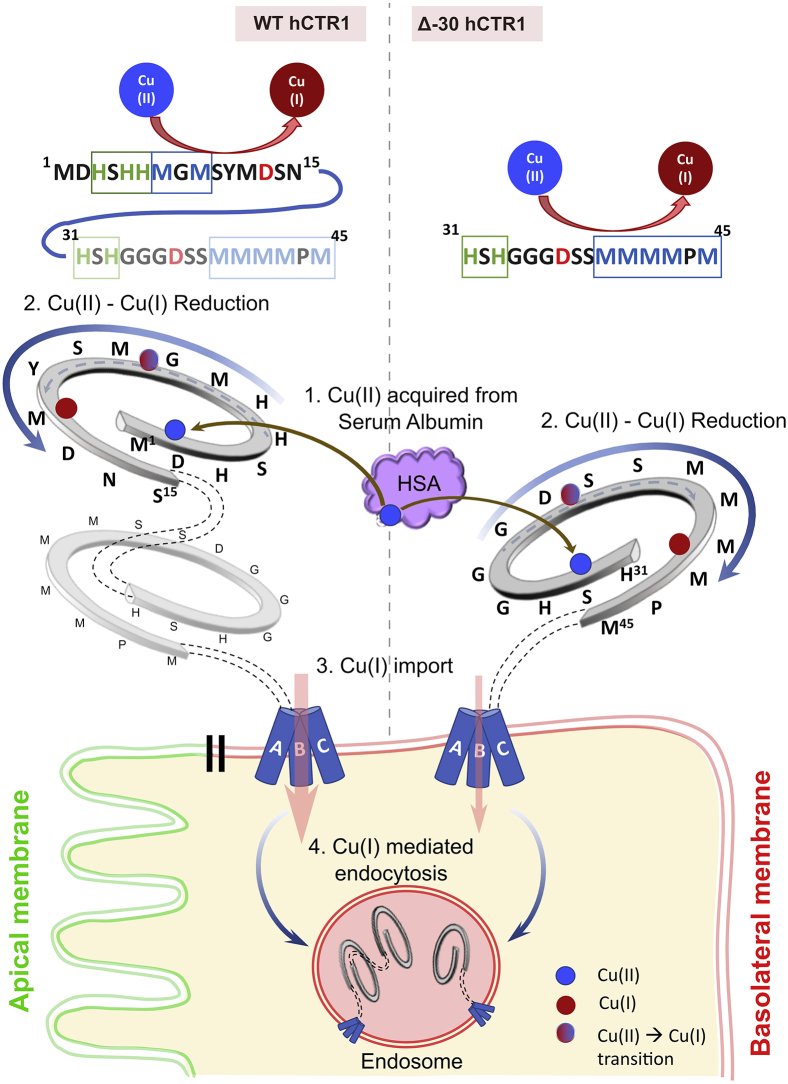
**Model depicting the correlation between the major functional aspects of the copper importer in both WT and Δ30-mutant constructs.** The WT-hCTR1 and Δ30-hCTR1, residing on the basolateral membrane of polarized epithelial cells, are capable of acquiring Cu(II) from Human Serum Albumin (HSA) by their histidine-rich stretches. Subsequently, the reduction of Cu(II) to Cu(I) possibly happens in participation with reducing agents. Aspartates facilitate this reduction and mediate the transfer of copper bound to the His-rich stretches in its +2 state to the adjacent methionines, which thereby binds and stabilizes Cu(I).This leads to import of Cu(I) and finally, Cu(I)-mediated endocytosis of the protein. Because of the complementary nature of His-Asp-Met clusters in the proximal and distal parts of the N-term, Δ30-hCTR1 can function like the full length WT, albeit the copper import property of the former is much lesser as compared with that of the later. hCTR1, human copper transporter-1.

To summarize, the His-Met clusters and the Asp residues on the distal part and the proximal part of the N-term exhibit complementarity. In the absence of the distal amino acid stretch harboring the first His-Met-Asp cluster, the second cluster can possibly perform the function of the first one in maintaining the redox state of copper that facilitates its uptake and subsequent endocytosis of hCTR1. Our findings provide an explanation of how the truncated version of hCTR1 that lacks O-linked glycosylation maintains functionality as a copper transporting-recycling protein (illustrated in Fig. 9).

## Discussion

Copper is indispensable for the maintenance of all eukaryotic life forms. Copper exists in two redox states, Cu(I) and Cu(II). Cu(II), owing to higher stability, is more abundant in nature. Cu(I), on the other hand, though biologically relevant, has a higher tendency to get oxidized. The long-standing conundrum that has existed in the field of biometals and, more specifically, in copper field is how Cu(II) is reduced to bioavailable Cu(I). The involvement of reductases has been hypothesized in the reduction mechanism. In yeast, the FRE family of metalloreductases facilitates copper acquisition by reducing copper from cupric [Cu(II)] to cuprous [Cu(I)] state. However, no clear and tangible mechanism underlying this reduction has been deciphered for mammalian cells. The STEAP family proteins (STEAP-2, STEAP-3, and STEAP-4) have been shown to reduce copper *in vitro* (59). Overexpression of these three STEAP proteins in HEK293T cells upregulates intracellular uptake of copper. Besides STEAPs, ascorbate has also been implicated in reducing Cu(II) to Cu(I) in the blood. Another interesting phenomenon that warrants a clear understanding is how the Cu(I) stays reduced during its process of getting transported inside the cell. Using a peptide-based model, Galler *et al.* (32) have recently shown that the trimeric arrangement of hCTR1 N-term promotes Cu(II) reduction and stabilization of the reduced form. However, the inference from this study has been limited by its *in vitro* nature and the utilization of just the ATCUN motif, encompassing the first four or six residues of the N-term of hCTR1.

In this study, we have taken a combinatorial approach, involving *in silico* and *in vivo* models to decipher the role of the N-term of hCTR1 in copper binding, maintaining its redox state and subsequent intracellular uptake. Further, we have delineated the relationship between copper uptake and endocytosis of the transporter.

We found that hCTR1 localized on the basolateral side of the polarized epithelial cell, MDCK-II. We further identified the compartment as the common recycling endosome where hCTR1 endocytoses upon copper treatment. Extrapolating this finding to an organ system, the amino-terminal of the transporter would be exposed to blood proteins transporting Cu(II), *e.g.*, albumin, under basal condition. Albumin, one of the most abundant proteins in blood serum, binds to Cu(II) and has been shown to transfer copper to the N-term of the copper transporter-1 in the presence of ascorbate (24, 52). We found that the N-terminal domain rich in His-Met not only binds to copper but also maintains the physiological redox state of the metal. Our model suggests that the histidine clusters in the trimeric hCTR1 bind to the Cu(II) and possibly increase its local concentration. Based upon studies from other groups, we hypothesize that ascorbate (soluble) and other membrane-bound reductases, *e.g.*, STEAP proteins convert Cu(II) to Cu(I) on the extracellular surface. Based upon our present study, we determined that the methionine clusters in combination with aspartates on the hCTR1 N-terminal domain facilitate reduction, bind, and stabilize the reduced copper state. Eventually, the Cu(I) enters the pore *via* its interaction with M^150^ and M^154^ triads situated on the entrance of the copper transporting pore. Cu(I) uptake in the cell induces endocytosis of the transporter, which regulates levels of copper import. The first methionine cluster, ^7^MGM^9^, serves as the primary coordinator for Cu(I), deletion of which leads to reduced copper uptake and subsequently abrogates hCTR1 endocytosis. Histidines, on the other hand, do not exhibit a direct interaction with Cu(I), and hence, its absence has no apparent effect on copper uptake or hCTR1 endocytosis. Interestingly, in limiting but physiological copper, the role of His motifs becomes more important to increase the local concentration of Cu(II) at the amino-terminal site of the protein for its subsequent reduction. We, for the first time, show the relevance of N-terminal aspartates in copper uptake and endocytosis of hCTR1. In our MD simulation model, we observed that aspartates in conjunction with histidines provide key ligation for Cu(II) binding. This is the possible reason for the relative higher frequency of aspartate residues in the region coded by exon 1 as compared with the rest of the three exons.

Our study supports a scenario according to which copper-induced endocytosis is a self-regulatory mechanism that limits copper uptake by the transporter. We found that the hCTR1 mutants that show reduced copper uptake also fail to endocytose. This observation is in agreement with the previous findings that mutating the methionines of the motif ^150^MXXXM^154^ either singly or in pairs inhibits copper uptake and also exhibits defective endocytosis in response to high extracellular copper (26). We hypothesized that the copper that is reduced in the extracellular milieu and is stabilized by the methionines of the amino terminus is subsequently relayed to the ^150^MXXXM^154 site^ for its eventual uptake.

We observed an interesting chasm in the conservation status of the proximal and distal parts of the amino-terminal of the transporter among various eukaryotic organisms. Based upon our copper uptake and endocytic assays in human CTR1, we determined that the proximal part can complement the function of the distal part in its complete absence. Some species (such as hamsters) lack the distal His-Met-Asp motifs (His^3–6^, Met^7–9^, and D13) (62). We hypothesize that the disposition of the His-Met-Asp motifs on the two regions of the amino-terminal provides flexibility among species in terms of their copper requirement and availability of copper in the environment where they thrive.

In the present study, we could not pinpoint whether the His-Met-Asp mutations had a direct effect on endocytosis or indirect effect through Cu(I) uptake. Though our study indicates that the cluster plays a role in stabilizing Cu(I) at the entrance of the CTR1 transmembrane pore, we cannot negate the possibility that the His-Met-Asp facilitates a endocytosis-ready conformation of the protein.

This study provides a clear understanding of the role of the amino-terminal of hCTR1 in maintaining a suitable redox state of copper that is key for its uptake. Further, we deciphered the differential participation of histidines, aspartates, and methionines in Cu(II) and Cu(I) binding. The proposed model is illustrated in Figure 9. hCTR1 has also been implicated in the binding and uptake of the anticancer drug, cisplatin, and other platinum complexes (63, 64). It will be important to determine if platinum drugs imported by hCTR1 follow a similar mechanism of binding to the amino-terminal, leading to its uptake and eventual endocytosis of the transporter.

## Experimental procedures

### Primers, plasmids, and antibodies

hCTR1 was cloned in p3XFLAG CMV10 vector (Sigma #E7658, a kind gift from Dr Rupasri Ain, CSIR-IICB) using HinDIII and EcoRI Restriction sites. Cytosolic myc-tagging and amino-terminal mutations were generated by following the Q5 Site-Directed Mutagenesis Kit (NEB #E0554) protocol. Primers for the site-directed mutagenesis were designed as per kit protocol and obtained from GCC biotech; their sequence details are provided in (Table S1). mEGFP-ATP7B and mKO2-ATP7A constructs were available in the lab. Following are the antibodies, used for different experiments: rabbit anti-FLAG M2 (CST #14793), mouse anti-FLAG (CST #8146), Goat anti-c-Myc(Novus #NB600-335), Mouse anti-Na/K ATPase (Invitrogen #MA3-929), anti-phalloidin 647 (Abcam #ab176759), Donkey anti-Rabbit IgG (H + L) Alexa Fluor 488 (Invitrogen #A-21206), Donkey anti-Mouse IgG (H + L) Alexa Fluor 568 (Invitrogen #A10037), Donkey anti-Goat IgG (H + L) Alexa Fluor 647 (Invitrogen #A21447), Rabbit anti-GAPDH (BioBharti #BB-AB0060), goat anti-rabbit IgG-HRP conjugated secondary antibody (BioBharti #BB-SAB01A). Plasmid isolations were done by QIAGEN plasmid mini kit (QIAGEN #27104).

### Cell lines and cell culture

MDCK-II cells were grown and maintained in Dulbecco’s Modified Eagle’s Medium (DMEM) (Sigma #D6429) supplemented with 10% Fetal Bovine Serum (FBS, Gibco #26140079), 1× Penicillin-Streptomycin (Gibco #15140122). For transfection, electroporation was performed using Nucleofector 2b and Amaxa kit V (Programme T023). After the electroporation, 3 × 10^5^ cells were grown in 0.4 μm inserts (Corning #3401). HEK293T cells were grown and maintained in DMEM supplemented with 10% FBS, 1× Penicillin-Streptomycin, 1× Amphotericin B (Thermo #15290026).

### Immunoblotting

Cells were grown on 60 mm dishes and transfected with respective plasmids containing WT and mutant hCTR1 constructs. Cell pellet was collected at 70% confluency. For lysate preparation, dry pellet was dissolved in 60 μl of lysis buffer [250 mM sucrose, 1 mM EDTA, 1 mM EGTA, 1× PBS as solvent, and 1× protease inhibitor cocktail (GCC Biotech)] and incubated on ice for 1 h. Then sonication was performed (four pulses; 5 s on phase and 30 s off phase; amplitude 100 mA) with a probe sonicator. Protein sample preparation was done by adding 4× loading buffer (Tris-HCl pH 6.81, 4% SDS, 10% β-mercaptoethanol, 20% glycerol, 0.02% Bromophenol Blue, and 8 M urea) to a final concentration of 1× and run on SDS-PAGE (10%) gels to separate proteins according to molecular mass. This was further followed by semidry transfer (Bio-Rad Trans-Blot SD Cell, Serial No. 221BR) of proteins onto nitrocellulose membrane (1620112, BioRad). After transfer, the membrane was blocked with 3% skimmed milk in 1× Tris-buffered saline (TBS) buffer pH 7.5 for 2 h at RT with mild shaking. Primary antibody incubation was done overnight at 4 °C following blocking and then washed with 1× TBST (0.01% Tween-20) for 10 min (three times). HRP-conjugated respective secondary antibody incubation was done for 2 h at RT, further washed, and signal was developed by ECL developer (170-5060, BioRad/1705062, BioRad) through chemiluminescence by Chemi Doc (BioRad). Protein marker was used- PAGEmark Tricolor Plus (G-biosciences #786-419).

### Copper treatments

A stock solution (10 mM) of copper chloride (SRL #92315) dissolved in water was used as a source of Cu(II) for copper treatment. For simulating the copper-chelated condition, a stock solution (2.5 mM) of TTM (Sigma #323446) dissolved in DMSO (Sigma #D2650) has been used. Direct Cu(I) was provided to the cells by treating with a mixture of CuCl_2_, L-ascorbic acid (Sigma-Aldrich #95209) and THPTA in ratio 1:2:2 in HBSS (Gibco #14025092). The latter two components were also dissolved in water, and a stock solution of 10 mM concentration each was prepared. All treatments were applied only on the basolateral side of the cells, if not mentioned otherwise. All treatment times are 1 h, if not mentioned otherwise.

### Transferrin internalization assay

For 633-Tf uptake assays, cells were starved for 60 min at 37 °C in HBSS containing 20 mM HEPES (standard buffer) and incubated for 60 min at 4 °C with 25 μg/ml 633-Tf in 1% BSA standard buffer. After that, temperature was shifted to 37 °C and, after the specified time, cells were immediately rinsed (with ice-cold HBSS) and fixed (ice-cold 4% PFA in PBS). After quenching PFA (with 50 mM NH_4_Cl in PBS) for 15 min, cells were permeabilized with 0.01% Triton X-100 in PBS for 7 min (65). Following this the cells are incubated with the corresponding primary antibodies for 2 h and then secondary antibodies again for 2 h in 1% BSA in PBS, and washed three times after each incubation.

To label BSE (basal sorting endosome) and CRE (common recycling endosome), Tf-633 was added to the basolateral side of the cells, growing on the membrane, for 5 min and 30 min, respectively, at 37 °C (25 μg/ml in 1% BSA standard buffer) (36).

### Immunofluorescence and microscopy

All the above-mentioned treatments were done after the cells reached polarization. After washing with ice-cold PBS (2 × 2 min), cells were fixed with 2% PFA in PBS for 20 min at room temperature (RT), followed by 20 min incubation with 50 mM ammonium chloride in PBS for quenching extra PFA. Next the cells were washed with PBS, and blocking was performed in 1% Bovine Serum Albumin (BSA, SRL #85171) in PBSS (0.075% saponin in PBS) for 20 min at RT. Primary antibody incubation was performed for 2 h at RT followed by PBSS washes (3 × 5 min). After that, incubation with the respective secondary antibodies was done for 2 h followed by five PBS washes. The membrane was mounted with the Fluoroshield with DAPI mountant (Sigma #F6057). All images were acquired with Leica SP8 confocal platform using oil immersion 63× objective (NA 1.4) and deconvoluted using Leica Lightning software.

### Image analysis

Images were analyzed in batches using ImageJ (66), image analysis software. For colocalization study, the Colocalization_Finder plugin was used. ROIs were drawn manually on the best z-stack for each cell. Manders’ colocalization coefficient (MCC) (67) was used for quantifying colocalization. Macro used in ImageJ is available in https://github.com/saps018/hCTR1-N-term/tree/main/colocalization.

### Statistical analysis

For statistical analysis and plotting, ggplot2 (68) and ggpubr (https://www.rdocumentation.org/packages/ggpubr/versions/0.1.1, accessed June 11, 2021) packages were used in R v-4.0.4 (69). Nonparametric tests for unpaired datasets (Mann–Whitney U test) were performed for all the samples.

### Determination of cellular copper concentrations by ICP-OES

MDCK-II cells were grown to polarization in 0.4 μm inserts in 6-well plates, and then differential copper treatments have been applied to the apical side and basolateral side of the polarized MDCK-II cells. Specific amount of copper chloride solution was added to the cell culture DMEM media and put that media into specific chambers of the polarized MDCK-II containing transwells (apical/basolateral), and incubation was done in the incubator for 1 h time duration for the treatment. Then they were washed with HBSS in a cold chamber and then incubated with TryplE Express (Gibco #12605028). Cells were harvested by scraping. Cells were pelleted down at 2500 rpm for 3 min and were washed five times with ice-cold DPBS (Gibco #14200075). The pellets were finally dissolved in DPBS and were counted by haemocytometer using Trypan Blue stain (Gibco #15250061). Further experiments were carried out with 2.5 × 10^6^ cells for each condition. Cell samples were digested for 16 h with 100 μl 65 % ICP-OES grade HNO_3_ at 95 °C. After digestion, samples were diluted in 5 ml of double distilled water and were syringe filtered through a 0.45-micron filter. Copper calibration is done by acid digestion of copper foil (procured from Alfa Aeasar) in 10 ml suprapure HNO_3_ for 1 h. (MWD conditions: Power = 400 W; Temperature = 100 °C; Hold time = 1 h). From the obtained solution, different solutions of varying copper strengths (50, 75, 100, 250, 500, 1000, 5000, 10,000 ppb) were prepared and were used for calibration. Copper concentration was determined using a Thermo Scientific inductively coupled plasma optical emission spectroscopy (ICP-OES) iCAP 6500.

### Strains, media, growth conditions for yeast complementation assay

For yeast complementation studies, *S. cerevisiae* *BY4742* (WT) strain (*MATα his3Δ1 leu2Δ0 lys2Δ0 ura3Δ0)* and an *S. cerevisiae* strain carrying an *yCTR1* deletion (Δ*yCTR1*) in the *BY4742* background purchased from Euroscarf (Oberursel) were used. YPD (yeast extract, peptone, and dextrose) medium was used for routinely maintaining both WT and deletion strains. For complementation assay, synthetic defined (SD) minimal media containing YNB (yeast nitrogen base), ammonium sulfate, and dextrose supplemented with histidine, leucine, lysine, and methionine (80 mg/L each) was used. Yeast transformations were carried out using the lithium acetate method (70). Human CTR1 was cloned in the yeast expression vector, *p416TEF* (as a positive control) and amino-terminal mutants were generated in the *hCTR1-p416TEF* construct by site-directed mutagenesis using Q5 Site-Directed Mutagenesis Kit (NEB #E0554) protocol. The *p416TEF* vector contains a *URA3* selection marker allowing growth in the absence of uracil. WT strain was transformed with an empty vector to allow its growth on SD-Ura (SD medium without uracil). Yeast transformants were selected and maintained on SD-Ura at 30 °C.

### *In vivo* functional complementation assay in *S. cerevisiae* by dilution spotting

Yeast transformants were grown overnight at 30 °C with shaking at 200 rpm in SD-Ura medium. The primary culture was used to inoculate secondary culture in the same selective medium and was allowed to grow at 30 °C till OD_600_ reached about 0.6. The cells were centrifuged, washed, and diluted in sterile water at OD_600_ = 0.2. Serial dilutions were then made with sterile water (OD_600_ = 0.2, 0.02, 0.002, 0.0002), and 10 μl of cell suspension from each was spotted on plates containing yeast extract, peptone, ethanol, and glycerol (YPEG plates). Plates were incubated at 30 °C for 3 to 5 days, and photographs were taken by Chemi Doc (BioRad). And to measure the growth rates of the yeast strain, we inoculated the yeast cultures grown up to saturation in SD-Ura liquid media to YPEG liquid media at a density of OD_600_ 0.2. Those cultures were then grown at 30 °C incubator for around 24 h, and growth density was measured spectroscopically after 0, 3, 4.5, 6, 7.5, 9, 10, 11, 12, 15, and 24 h. Growth curves of yeasts expressing WT and mutant hCTR1 in YPEG culture media were generated over that 24-h period. Yeast growth rates (ΔOD_600_/hour) were calculated from linear exponential growth phase between 3 and 12 h time points. Average growth rates were calculated from four different independent experiments.

### MD simulation

#### A. System setup for simulation

The starting structure of the hCTR1 protein is an electron crystallography structure provided by Professor Vinzenz Unger, Department of Molecular Biosciences, NorthWestern University (45). Only the extracellular N-terminal domain of the three monomers of the protein (residues 1–67) is considered for the simulations (system setup shown in Fig. S3*A*). The protein and water are represented using the CHARMM36 force field (71). The Cu(II)/Cu(I) are represented as virtual site models in an octahedral and tetrahedral geometry, respectively (47, 48). Each of the N-term-copper systems was solvated by ∼16,000 TIP4P water molecules in a box of dimensions 80 × 80 × 80 Å3. The physiological concentration (150 mM) of Na+ and Cl-ions along with extra Na+ ions was used to neutralize the system. Simulations were performed using molecular dynamics software GROMACS 2019.6 (https://doi.org/10.5281/zenodo.2424486).

#### B. Equilibration and simulation

Initially, each system is energy minimized using the steepest descent method (1) for 10,000 steps, followed by heating it to 300 K in 200 ps using Berendsen thermostat and barostat (72) with a coupling constant of 0.5 ps each. Restraints of 25 kcal/mol/Å^2^ are applied on heavy atoms during the heating process. Thereafter, equilibration is carried out for 2 ns at constant temperature (300 K) and pressure (1 bar) without any restraints using the same thermostat and barostat with coupling constants of 0.2 ps each. The last 100 ps of NPT simulation is used to calculate the average volume, which is used in all simulations going forward. Unrestrained NVT equilibration for 200 ns at temperature 300K is carried out using the velocity-rescale thermostat (73) with coupling constant of 0.1 ps. During the simulation, LINCS algorithm (74) is used to constrain all the bonds, and Particle Mesh Ewald (PME) method (75) is used for electrostatics. The distance cutoffs for the van der Waals (vdW) and electrostatic long-range interaction are kept at 12 Å. The time step for each simulation is taken to be 1 fs.

#### C. Free-energy calculation using metadynamics

The equilibrated N-terminal domain is initially simulated for 5 ns in the presence of an unbound copper ion. Free-energy calculations are performed after the Cu(I)/Cu(II) binds to the N-terminal domain. To calculate the binding free energy of the ion in both of its oxidation states, well-tempered metadynamics (76) simulations are performed after equilibration using distvec (49) (Fig. S4*B*) and native contacts (N_c_) (Fig. S4*C*) as collective variables. We performed a long (∼150 ns). metadynamics simulation with a hill height of 0.2 kJ/mol and a bias factor of 10 and hills deposition rate of 2 ps. Gaussian widths for distvec and native contacts are taken to be 0.6 Å and 5, respectively. An upper wall restraint is applied at 45 degrees on the angle between two vectors, as shown in Fig. S4*B* of SI. For free-energy calculations, PLUMED 2.6 (77). is used along with GROMACS. The system size and run lengths of all the systems are provided in the Fig. S4*D* of SI.

### Quantum mechanical (QM) calculations

The electronic structure calculations are performed using first-principles DFT (78, 79) as implemented in the Quantum ESPRESSO package (80). To describe the exchange-correlation energy functional, a generalized gradient approximation is used as given by Perdew, Burke, and Ernzerhof (81). Kohn–Sham wave functions are expanded using a plane wave basis set, with wave function and charge density cutoffs of 50 and 500 Ry, respectively. Valence electrons are described by Projector Augmented Wave (PAW) approach (82). The calculations are performed at a single k-point, Gamma—the center of the Brillouin zone. The occupation numbers are treated according to the gaussian scheme with a broadening of 0.001 Ry. Atomic coordinates are relaxed using the Broyden–Fletcher–Goldfarb–Shanno (BFGS) scheme, with a force convergence criterion of 0.001Ry/Bohr (83, 84, 85) (https://www.ams.org/journal-terms-of-use, accessed December 2, 2021). A vacuum spacing of 20 Å was introduced along all the three directions, so as to reduce interactions between the periodic images. Spin-polarized calculations are performed in order to capture spin states of copper atoms correctly.

### Synthesis of tris-(3-hydroxypropyl triazolyl methyl)-amine (THPTA)

The synthesis and characterization of THPTA have been described in detail in this paper (60). Production of correct THPTA was validated by ^1^H NMR (400 MHz, DMSO-D_6_) δ ppm 1.93 to 1.99 (m, 6H), 3.40 to 3.43 (m, 6H), 3.62 (s, 6H), 4.39 to 4.42 (t, 6H), 4.67 to 4.70 (t, 3H), 8.03 (s, 3H) and ^13^C NMR (400 MHz, DMSO-D_6_) δ ppm 32.99, 46.58, 47.10, 57.50, 123.99, 143.42.

### Electron paramagnetic resonance

EPR data were recorded with an EMX MICRO X, Bruker spectrometer, operating at a microwave frequency of approximately 9.75 GHz. Spectra were recorded using a microwave radiation power of 10 mW across a sweep width of 2000 G (centered at 2200 G) with modulation amplitude of 10 G. Experiments were carried out at 100 K using a liquid nitrogen cryostat.

EPR samples were prepared from 5 mM CuCl_2_, 10 mM ascorbate, and 10 mM THPTA solutions dissolved in water in order to distinguish between the oxidation states of copper in the presence of ascorbate and/or THPTA. Samples were frozen in a quartz tube after addition of 10% glycerol as a cryoprotectant and stored in liquid nitrogen until used.

### UV–Visible spectroscopy

UV-Vis spectra were recorded at room temperature on Cary 8454 Agilent spectrophotometer, over the spectral range 300 to 800 nm, using the 1 cm-path-length quartz cuvettes. 20 mM CuCl_2_ were mixed with different equivalents (ranging from 0.5 to 2 equivalence) of ascorbate and two equivalences of THPTA in water, and spectra were recorded at two different time points, 0 min and 30 min.

### Sequence alignment and conservation status

CTR1 sequences all over the species were obtained from NCBI. Exon composition of chordate *CTR1* was obtained from Ensembl (https://www.ensembl.org/) (86). Sequence alignment file was created in Clustal Omega (87, 88). For sequence visualization, Jalview 2.11.1.4 was used (89). Amino acid composition details were created using python script. Protter was used for preparing a representative image of CTR1 (90). Python codes used in exon analysis are available in https://github.com/saps018/hCTR1-N-term/tree/main/Exon%20analysis.

## Data availability

All data described in this manuscript are contained within the manuscript.

## Supporting information

This article contains supporting information.

## Conflict of interest

The authors declare that they have no conflicts of interest with the contents of this article.
