# Supplementary material for: Copper(II) import and reduction are dependent on His-Met clusters in the extracellular amino terminus of human copper transporter-1
Source: J Biol Chem. 2022 Jan 26;298(3):101631. doi: 10.1016/j.jbc.2022.101631 (PMC8867124; doi:10.1016/j.jbc.2022.101631)
Supplement: Supplemental Figures S1–S7 and Table S1 [file mmc1.pdf]

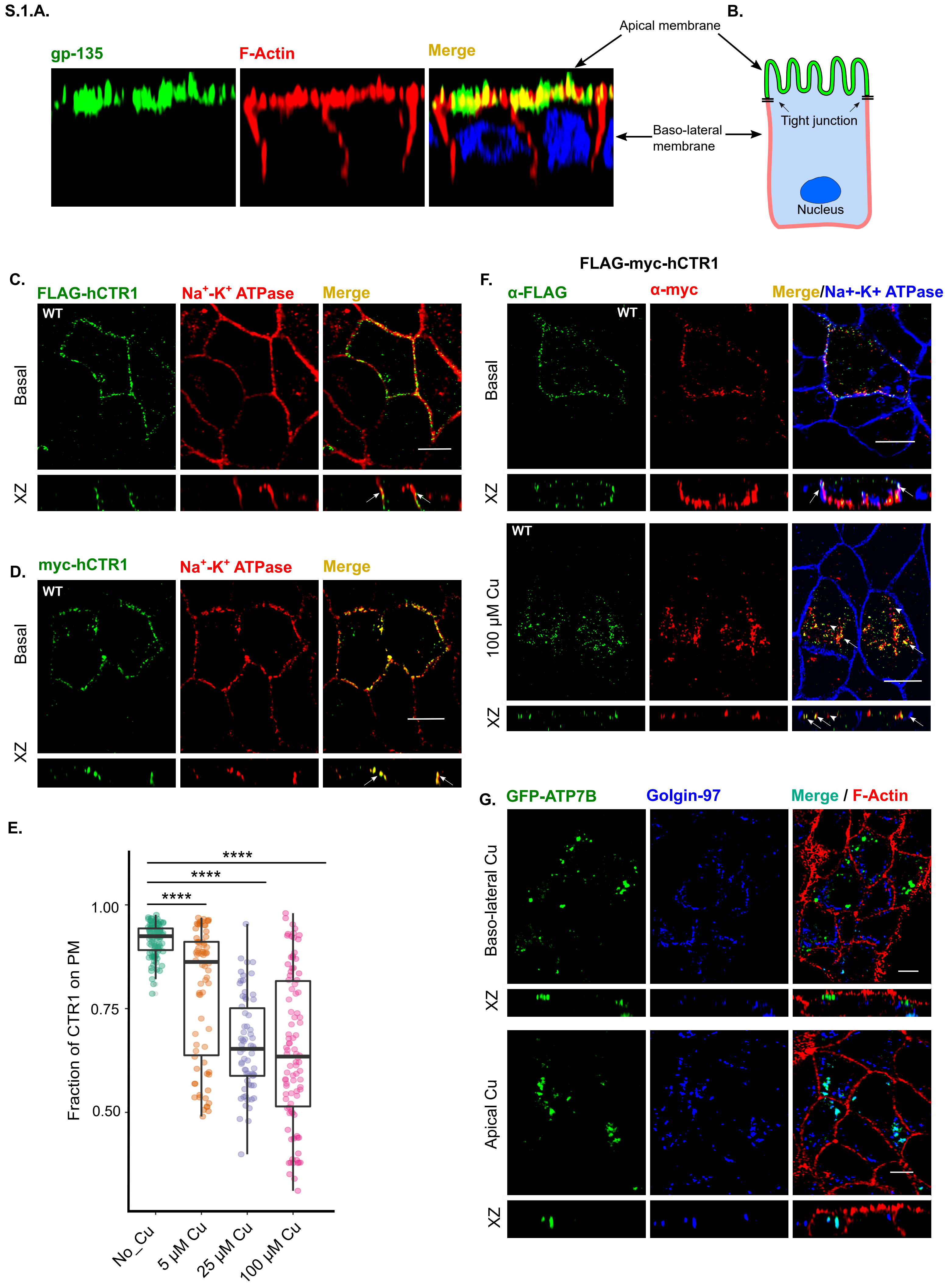

S.1.A.
B.
gp-135
F-Actin
Merge
Apical membrane
Baso-lateral membrane
Tight junction
Nucleus
C.
FLAG-hCTR1
Na+-K+ ATPase
Merge
WT
Basal
XZ
D.
myc-hCTR1
Na+-K+ ATPase
Merge
WT
Basal
XZ
E.
Fraction of CTR1 on PM
1.00
0.75
0.50
****
****
****
No_Cu
5 µM Cu
25 µM Cu
100 µM Cu
FLAG-myc-hCTR1
F.
α-FLAG
α-myc
Merge/Na+-K+ ATPase
WT
Basal
XZ
WT
100 µM Cu
XZ
G.
GFP-ATP7B
Golgin-97
Merge / F-Actin
Baso-lateral Cu
XZ
Apical Cu
XZ

S.2.

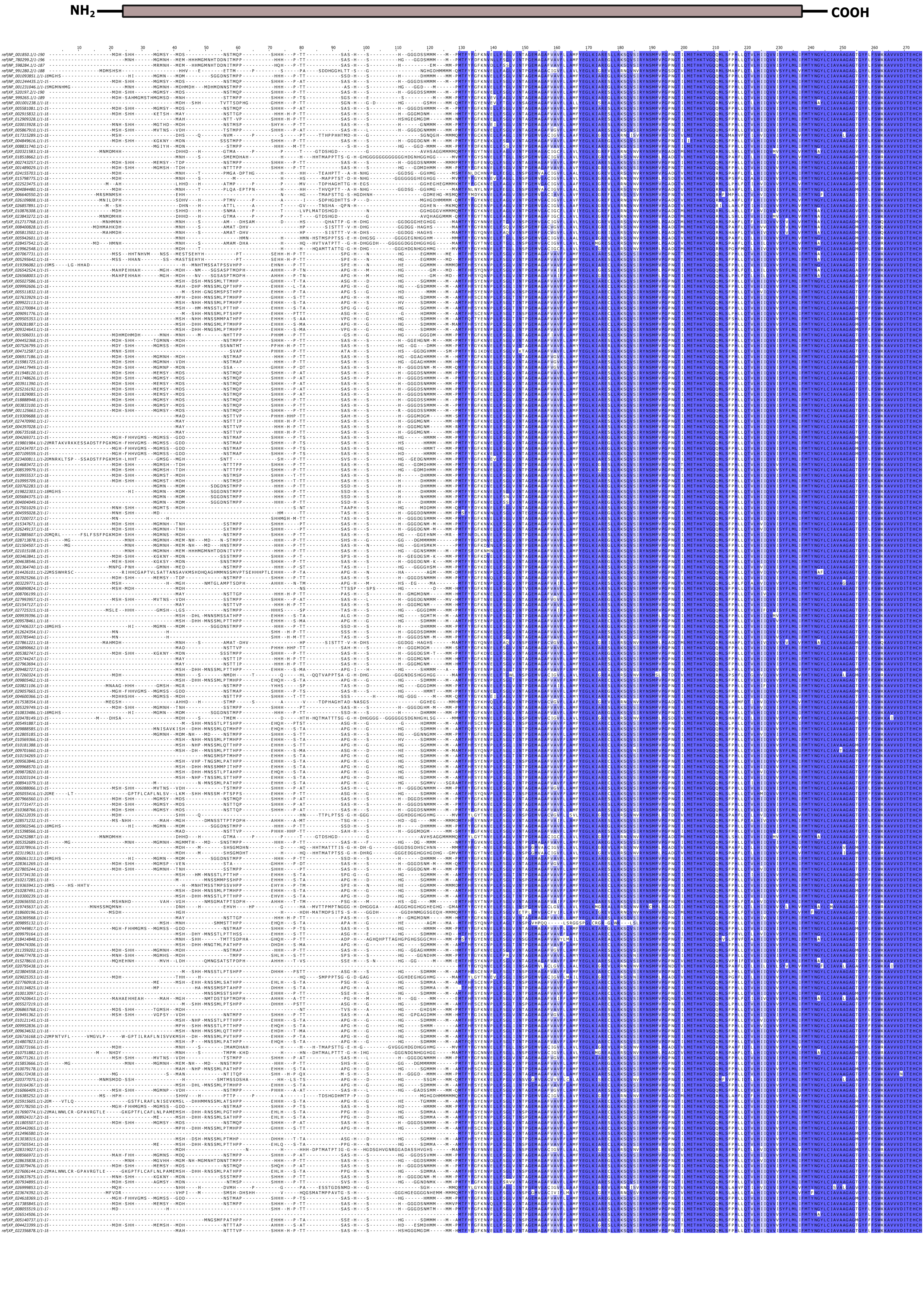

S.3.A.

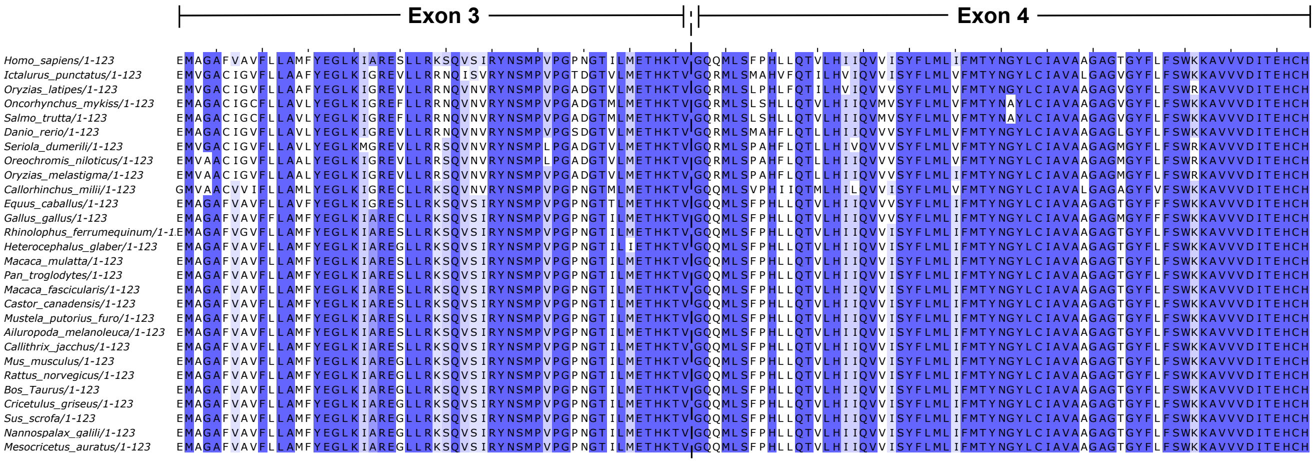
Exon 3
Exon 4
Homo_sapiens/1-123
Ictalurus_punctatus/1-123
Oryzias_latipes/1-123
Oncorhynchus_mykiss/1-123
Salmo_trutta/1-123
Danio_rerio/1-123
Seriola_dumerili/1-123
Oreochromis_niloticus/1-123
Oryzias_melastigma/1-123
Callorhinchus_milii/1-123
Equus_caballus/1-123
Gallus_gallus/1-123
Heterocephalus_glaber/1-123
Macaca_mulatta/1-123
Pan_troglodytes/1-123
Macaca_fascicularis/1-123
Castor_canadensis/1-123
Mustela_putorius_furo/1-123
Ailuropoda_melanoleuca/1-123
Callithrix_jacchus/1-123
Mus_musculus/1-123
Rattus_norvegicus/1-123
Bos_Taurus/1-123
Cricetulus_griseus/1-123
Sus_scrofa/1-123
Nannospalax_galili/1-123
Mesocricetus_auratus/1-123

B.

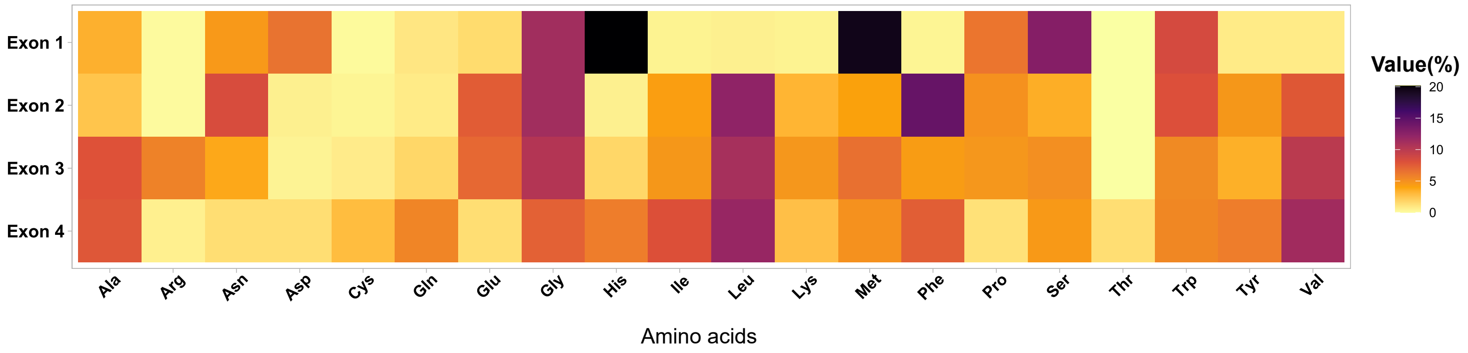
Exon 1
Exon 2
Exon 3
Exon 4
Value(%)
20
15
10
5
0
Ala
Arg
Asn
Asp
Cys
Gln
Glu
Gly
His
Ile
Leu
Lys
Met
Phe
Pro
Ser
Thr
Trp
Tyr
Val
Amino acids

C.

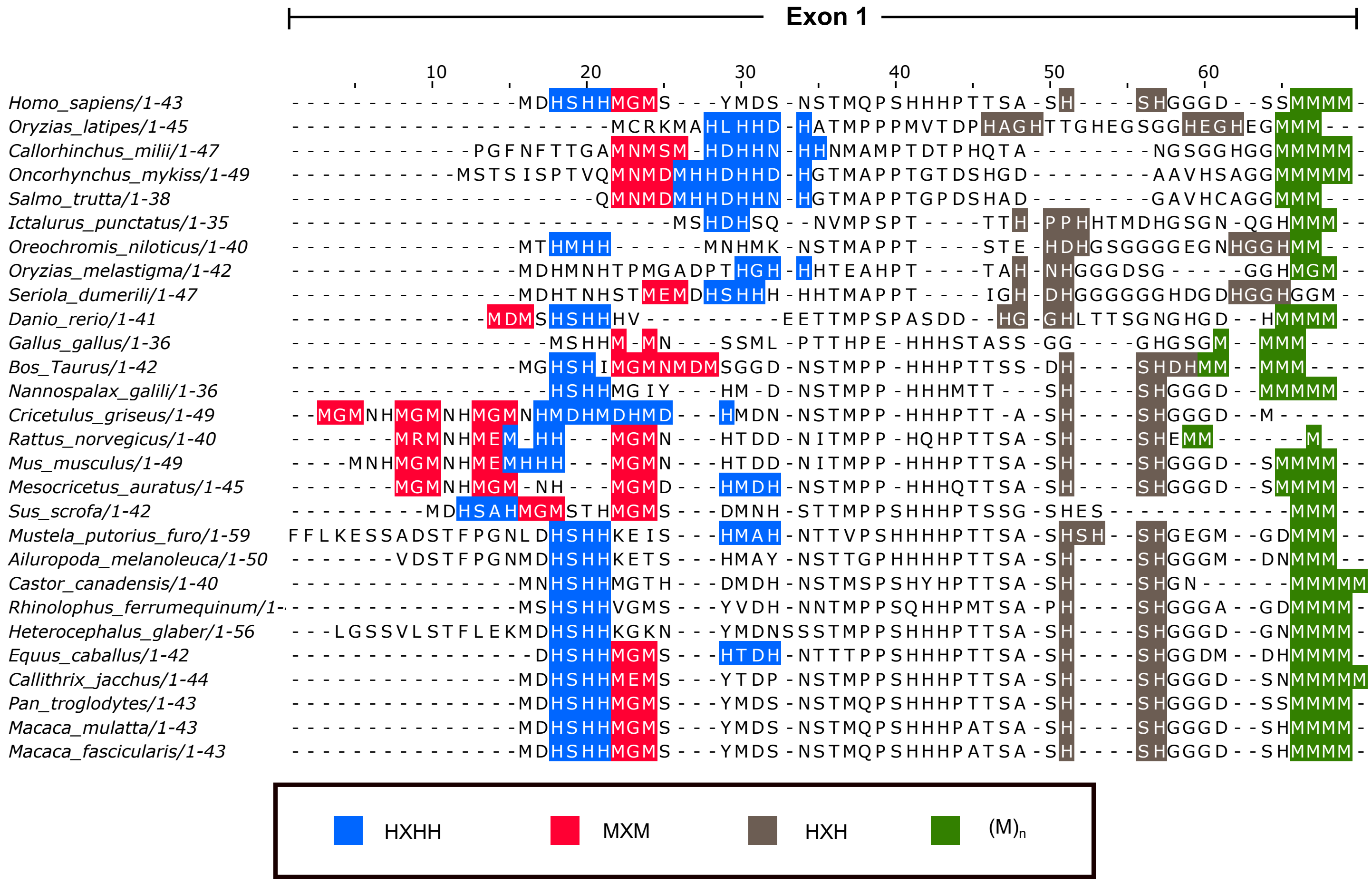
Exon 1
10
20
30
40
50
60
Homo_sapiens/1-43
Oryzias_latipes/1-45
Callorhinchus_milii/1-47
Oncorhynchus_mykiss/1-49
Salmo_trutta/1-48
Ictalurus_punctatus/1-35
Oreochromis_niloticus/1-40
Oryzias_melastigma/1-42
Seriola_dumerili/1-47
Danio_rerio/1-41
Gallus_gallus/1-36
Bos_Taurus/1-42
Nannospalax_galili/1-36
Cricetulus_griseus/1-49
Rattus_norvegicus/1-40
Mus_musculus/1-49
Mesocricetus_auratus/1-45
Sus_scrofa/1-42
Mustela_putorius_furo/1-59
Ailuropoda_melanoleuca/1-50
Castor_canadensis/1-40
Heterocephalus_glaber/1-56
Equus_caballus/1-42
Callithrix_jacchus/1-44
Pan_troglodytes/1-43
Macaca_mulatta/1-43
Macaca_fascicularis/1-43
HXHH
MXM
HXH
(M)n

S.4.A.

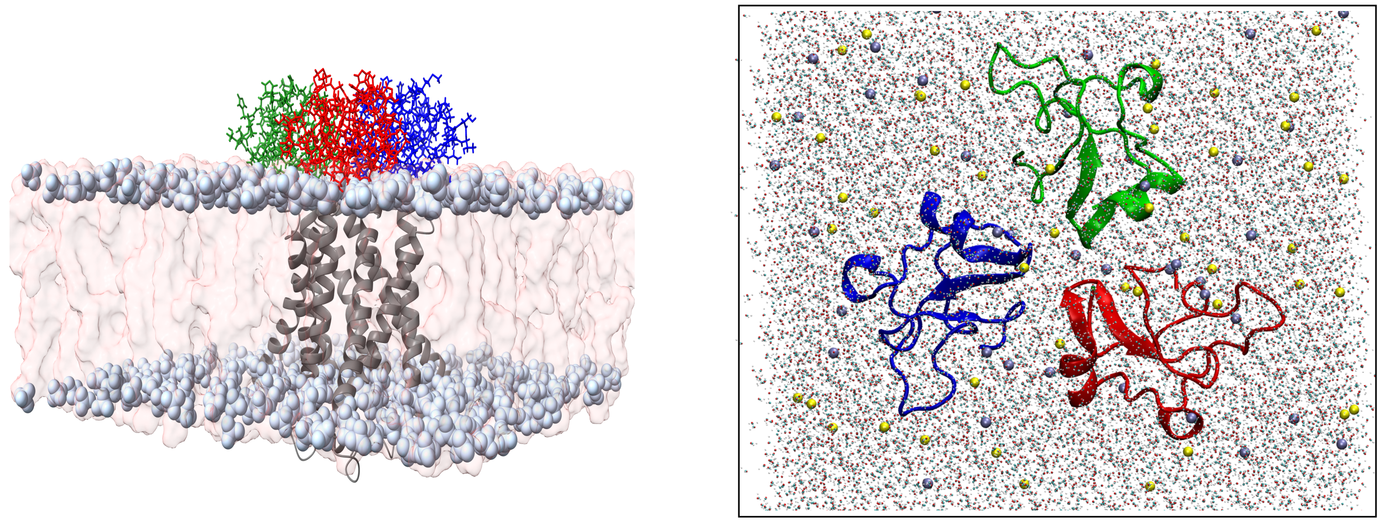

B.

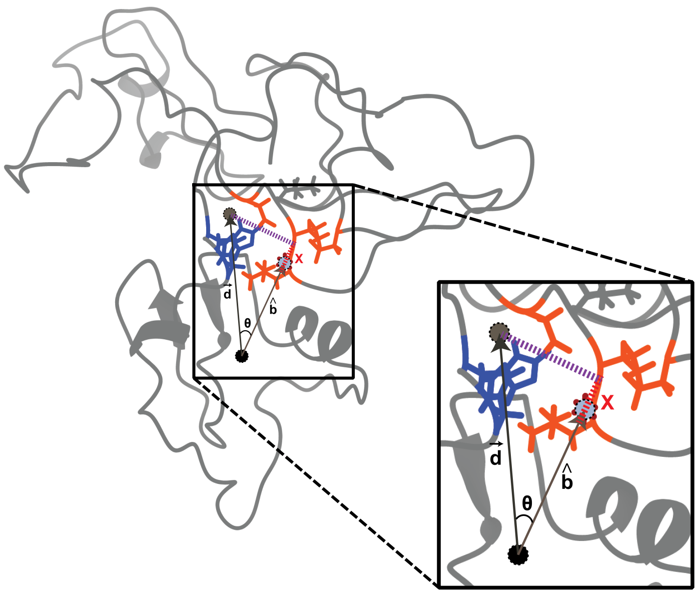

C.

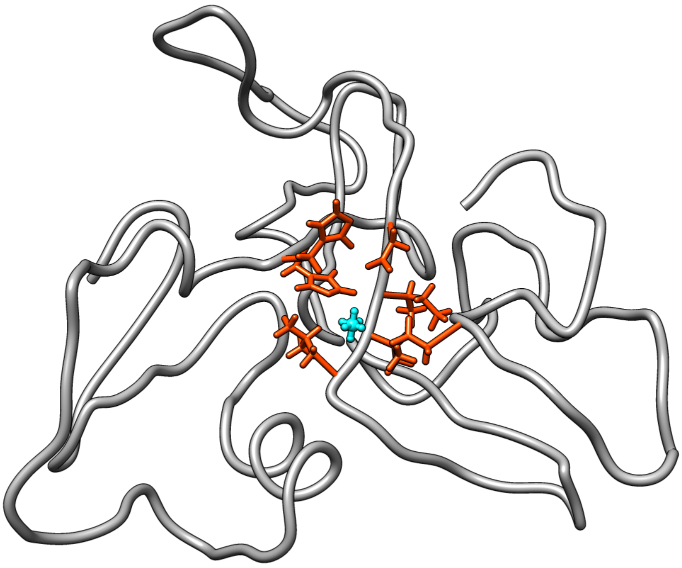

D.

System	Runtime (ns)	System Size (Number of atoms)
Cu(II)-Nterm (run 1)	189.94	66252
Cu(II)-Nterm (run 2)	178.49	66252
Cu(II)-Nterm (run 3)	191.66	66252
Cu(I)-Nterm (run 1)	116.4	66275
Cu(I)-Nterm (run 2)	157.8	66275
Cu(I)-Nterm (run 3)	225.64	66275

S.5.

After MD	After QM

1.

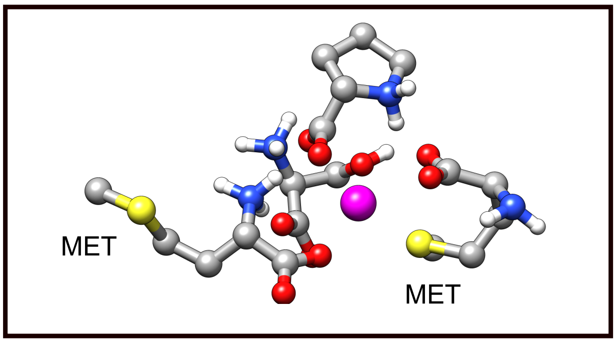

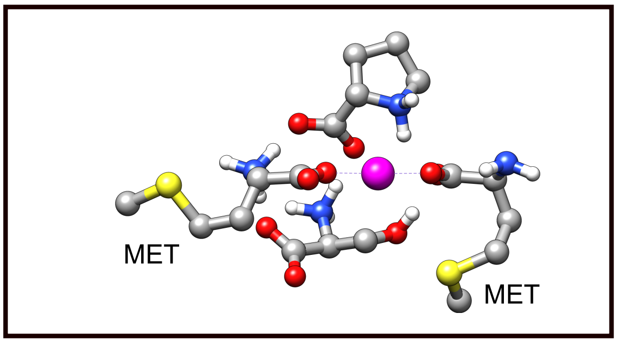

2.

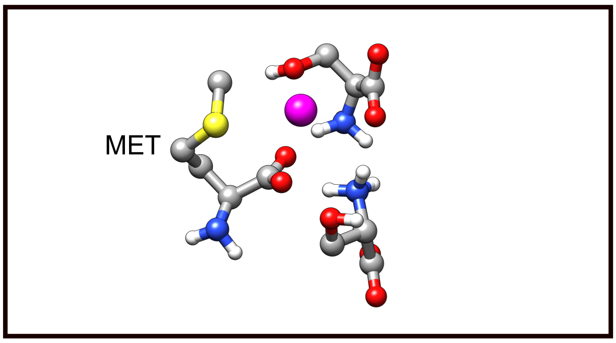

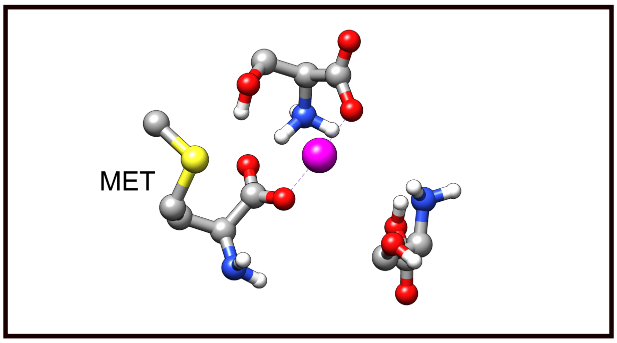

3.

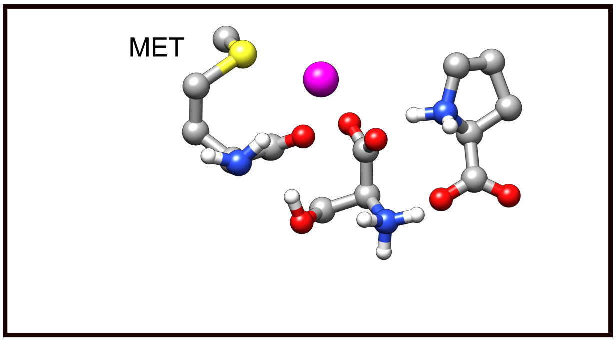

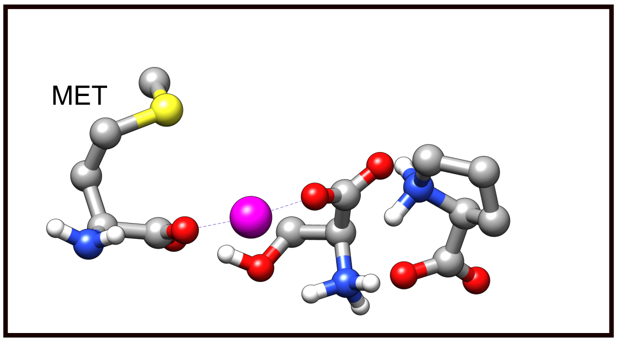

4.

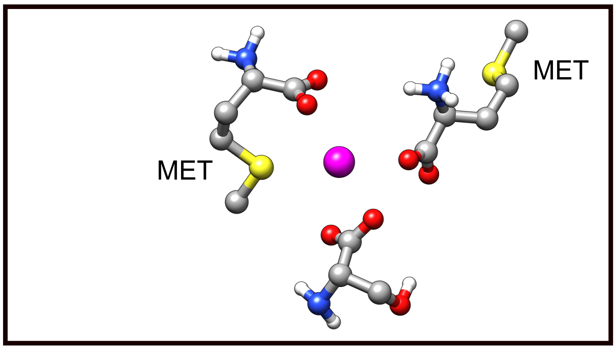

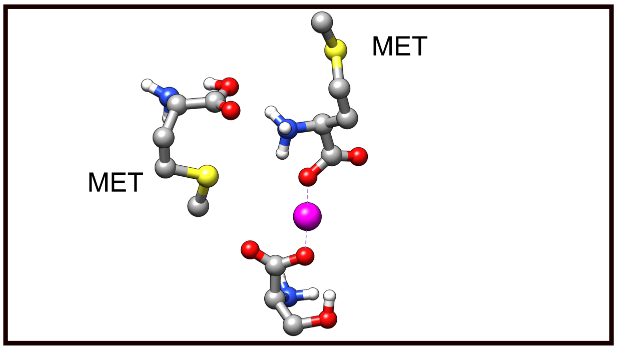

5.

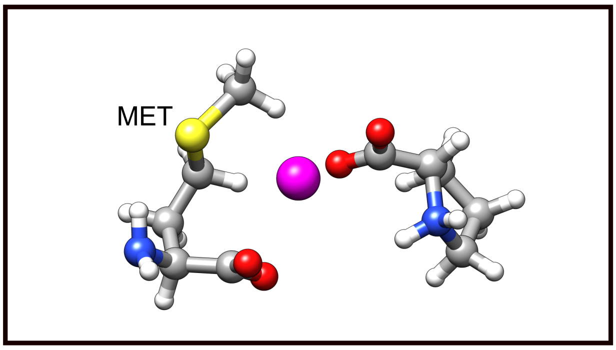

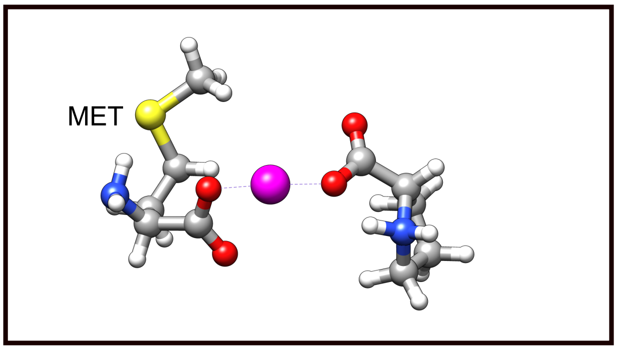

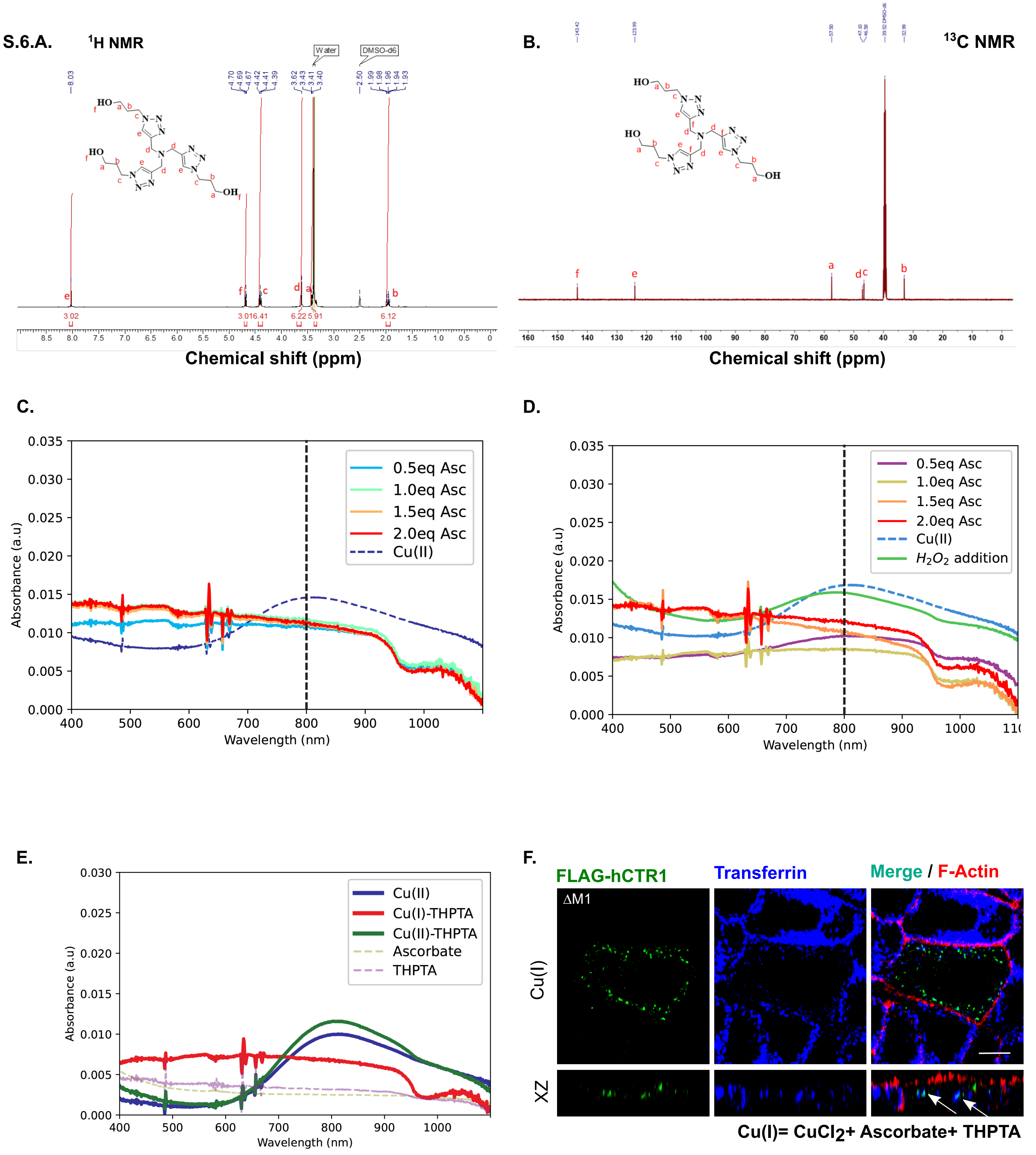

S.6.A.
1H NMR
Water
DMSO-d6
Chemical shift (ppm)
B.
13C NMR
Chemical shift (ppm)
C.
0.5eq Asc
1.0eq Asc
1.5eq Asc
2.0eq Asc
Cu(II)
Absorbance (a.u)
Wavelength (nm)
D.
0.5eq Asc
1.0eq Asc
1.5eq Asc
2.0eq Asc
Cu(II)
H2O2 addition
Absorbance (a.u)
Wavelength (nm)
E.
Cu(II)
Cu(I)-THPTA
Cu(II)-THPTA
Ascorbate
THPTA
Absorbance (a.u)
Wavelength (nm)
F.
FLAG-hCTR1
Transferrin
Merge / F-Actin
ΔM1
Cu(I)
XZ
Cu(I)= CuCl2+ Ascorbate+ THPTA

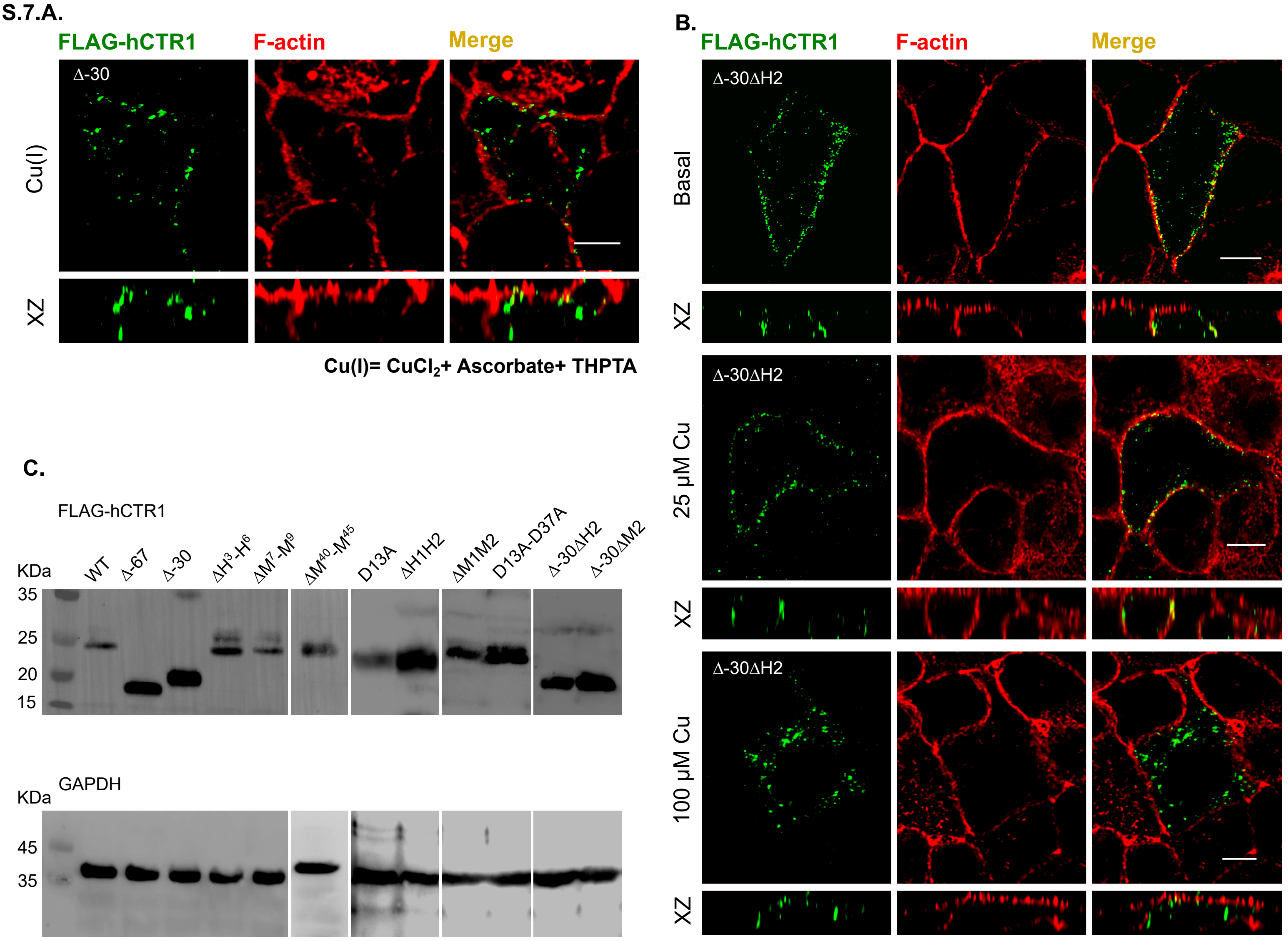

S.7.A.
FLAG-hCTR1
F-actin
Merge
Δ-30
Cu(I)
XZ
Cu(I)= CuCl2+ Ascorbate+ THPTA
B.
FLAG-hCTR1
F-actin
Merge
Δ-30ΔH2
Basal
XZ
Δ-30ΔH2
25 μM Cu
XZ
Δ-30ΔH2
100 μM Cu
XZ
C.
FLAG-hCTR1
KDa
WT
Δ-67
Δ-30
ΔH3-H6
ΔM7-M9
ΔM40-M45
D13A
ΔH1H2
ΔM1M2
D13A-D37A
Δ-30ΔH2
Δ-30ΔM2
35
25
20
15
GAPDH
KDa
45
35

Supplementary Table

Primer Name	Sequence (5′-3′)
**HinDIII-hCTR1_FP**	ACTTTCCCAAGCTTATGGATCATTCCCACCATATGG
**EcoRI-hCTR1_RP**	AGTTTCCGGAATTCTCAATGGCAATGCTCTGTGATA
**Δ-67 hCTR1_FP**	TTGGTGATCAATACAGCTG
**Δ-67 hCTR1_RP**	AAGCTTGTCATCGTCATC
**Δ-30 hCTR1_FP**	CACTCCCATGGTGGAGGA
**Δ-30 hCTR1_RP**	AAGCTTGTCATCGTCATCC
**ΔH3-H6_FP**	ATGGGGATGAGCTATATG
**ΔH3-H6_RP**	ATCCATAAGCTTGTCATC
**ΔM7-M9_FP**	AGCTATATGGACTCCAAC
**ΔM7-M9_RP**	ATGGTGGGAATGATCCATAAG
**D13A_FP**	AGCTATATGGCTTCCAACAGTACCATG
**D13A_RP**	CATCCCCATATGGTGGGA
**ΔM40-M45_FP**	ACCTTCTACTTTGGCTTTAAGAATG
**ΔM40-M45_RP**	GCTGCTGTCTCCTCCACC
**D37A_FP**	GGTGGAGGAGCCAGCAGCATG`
**D37A_RP**	ATGGGAGTGTGAGGCTGAA
**ΔH31-H33_FP**	GGTGGAGGAGACAGCAGC
**ΔH31-H33_RP**	TGAGGCTGAAGTGGTTGG
**FLAG-hCTR1_XbaI_FP**	TGCTCTAGAATGGACTACAAAGACCATGACGGT
**FLAG-hCTR1_BamHI_RP**	CGCGGATCCTCAATGGCAATGCTCTGTGATATCCAC
**Myc_FP**	TTCTGAAGAAGATCTTATGGAGACACACAAAAC
**Myc_RP**	ATAAGTTTTTGTTCGATGGTTCCATTTGGTCC
