# Supplementary material for: Copper(II) import and reduction are dependent on His-Met clusters in the extracellular amino terminus of human copper transporter-1
Source: J Biol Chem. 2022 Jan 26;298(3):101631. doi: 10.1016/j.jbc.2022.101631 (PMC8867124; doi:10.1016/j.jbc.2022.101631)
Supplement: Supplemental Figures and Table Legends [file mmc2.docx]

**Legends to Supplementary Figures:**

**S.1. (A)** Representative image of polarized MDCK-II cell line after fixation and staining. (Upperside is the Apical side, stained with gp-135 (also known as podocalyxin); (Green- gp-135, Red- F-Actin, Blue- DAPI). After the polarization, cells attain height ~ 12-15 μm and form distinct apical and basolateral sides separated by gap junctions. **(B)** Representation of a polarized MDCK-II cell. **(C)** Wild-type (WT) FLAG-hCTR1 (green) under basal condition colocalizes with Na, K ATP-ase (red), white arrow in the XZ section exhibits basolateral localization of hCTR1. **(D)** Wild-type (WT) Myc-hCTR1 (green) under basal condition colocalizes with Na,K ATP-ase (red), white arrow in the XZ section exhibits basolateral localization of hCTR1. **(E)** Fraction of WT-hCTR1 colocalization with membrane marker F-actin, demonstrated by box plot with jitter points. The box represents the 25–75th percentiles, and the median in the middle. The whiskers show the data points within the range of 1.5× interquartile range (IQR) from the 1st and 3rd quartile. ****P<0.0001 (non-parametric Mann–Whitney U test/Wilcoxon rank-sum test). Sample size (n) for Basal: 100, 5 uM Cu: 74, 25 uM Cu: 66, 100 uM Cu: 101. **(F)** Flag-Myc-hCTR1 colocalizes with Na, K ATPase under basal copper condition (upper panel) White arrows on the XZ section denotes the lateral colocalization of the two proteins. At high copper Flag-Myc-hCTR1 endocytoses (bottom panel). Yellow signal denotes colocalization of the unprocessed hCTR1 containing both tags (indicated by arrow); Red signal belongs exclusively to processed hCTR1 lacking a part of the N-terminus (indicated by arrowhead) **(G)** Transiently transfected GFP-ATP7B shows trafficking towards the apical membrane (upper panel) upon copper treatment in the basolateral chamber whereas the protein shows no trafficking from Golgi during copper treatment only on the apical side (lower panel) [In all the conditions, cells are polarized MDCK-II, XZ section shows the orthogonal sections of all the stacks, green- GFP-ATP7B, blue- Golgin-97 and red- F-actin; Cu treatment- 100μM; scale bar- 5μm].

**S.2.** Sequence alignment of CTR1s across the species. Upper bar shows the direction of amino acid sequences from amino-terminal (NH_2_) to carboxylic-terminal (COOH). Conserved residues are marked by blue colour. The intensity of the blue colour is proportional to the conservation status of a residue.

**S.3. (A)** Sequence alignment of the last two exons (Exon 3 and Exon 4) of chordata CTR1s. Conserved residues are marked by blue colour. The intensity of the blue colour is proportional to the conservation status of a residue. **(B)** Heat map showing the percentages of the different amino acids encoded by each of the four chordate CTR1 exons. **(C)** Multiple histidine and methionine clusters on Exon1 of chordate CTR1 are shown with colour coded selection.

**S.4. (A)** hCTR1 is a trimeric membrane protein (transmembrane regions depicted as ribbons in grey) that is shown to be embedded in a POPC lipid bilayer (shown as a surface with phosphate head groups depicted as spheres) (left panel). The simulation setup in this work comprises only the extracellular N-terminal region of the protein in water. Na+ ions are shown in yellow and Cl- ions in mauve.[top view of the system in (right panel)]. The trimeric conformation of the N-terms is maintained by putting position restraints on the heavy atoms of the last residues of each monomer. **(B)** The schematic description of the distvec collective variable which is mathematically specified as the body-fixed distance to the ligand/ion of interest. In the figure the protein is shown as a grey silhouette, the center of mass (c.o.m) of the protein is shown by the black circle, the c.o.m of the binding region is given by the violet circle, and the c.o.m of the Cu(II)-octahedral dummy model is depicted by the light blue circle. The residues marked in deep blue and orange are the histidine-rich and histidine-deficient halves of the binding site respectively, which are used to body-fix the coordinate axes **(C)** The Native contact (𝑵𝑵𝒄𝒄) is defined by the spatial proximity of groups of atoms 𝒈𝒈𝑨𝑨 and 𝒈𝒈𝑩𝑩 in the native state. The Cu(II) virtual site model (shown in cyan) constitutes 𝒈𝒈𝑨𝑨 while the heavy atoms of part of the protein that resides within 5.5 Å from the c.o.m (center of mass) of the copper dummy model constitute 𝒈𝒈𝑩𝑩 (shown in orange). **(D)** The system size and simulation lengths for the metadynamics simulation for all the systems are given in Fig 3D.

**S.5.** Comparison of representative structures between MD simulations (left panel) and QM calculations (right panel) indicates very low structural deviation in the Cu(I) coordination complexes obtained through both techniques.

**S.6.(A)**1^H^ NMR of 3,3',3''-(4,4',4''-(nitrilotris(methylene))tris(1H-1,2,3-triazole-4,1-diyl))tris(propan-1-ol)(THPTA); In X-axis chemical shift is indicated **(B)**13^C^ NMR of 3,3',3''-(4,4',4''-(nitrilotris(methylene))tris(1H-1,2,3-triazole-4,1-diyl))tris(propan-1-ol)(THPTA). In X-axis chemical shift is indicated. **(C)** UV-Vis Spectra shows a wide band for Cu(II) with an absorption maximum at 800 nm. 20mM CuCl2 in water is treated with 2 equivalence of THPTA and varying equivalence of ascorbate ranging from 0.5 to 2 equivalence at two different time points, 0 min **(C)** and 30 min **(D)** respectively. **(D)** Addition of an oxidising agent, H2O2 to the ascorbate+THPTA+CuCl2 (2:2:1) mixture, at the end of 30 mins causes the peak for Cu(II) to return, indicating the reoxidation of Cu(I). **(E)** The peak for Cu(II) at 800 nm retains itself when 40mM THPTA is added to 20mM CuCl2 solution (Cu(II)-THPTA), but in presence of both 40mM ascorbate and 40mM THPTA, the peak vanishes indicating the formation of Cu(I)-THPTA, which is not UV-Vis active. **(F)** ΔM1 (ΔM7-M9) endocytoses when treated with ascorbate +THPTA + CuCl2 in 2:2:1 ratio {readymade source of Cu(I)} and colocalizes with the basolateral sorting endosomes and common recycling endosomes (marked by post-30 min internalization of Transferrin-Alexa 633). [The cell is polarized MDCK-II, XZ section shows the orthogonal sections of all the stacks, green- FLAG-hCTR1, Blue- Transferrin and red- F-actin; (200μM ascorbate + 200μM THPTA + 100μM CuCl2) treatment on the basolateral chamber of the transwell, scale bar- 5 μm].

**S.7. (A)** Δ-30 exhibits endocytosis when treated with the ready source of Cu(I). **(B)** Δ-30ΔH2 mutant resides on the PM under basal (upper panel) conditions, 25μM Cu treatment (middle row) also fails to induce endocytosis of the same. In response to 100μM Cu (lower panel) however, Δ-30ΔH2 endocytoses. [In all the conditions, cells are polarized MDCK-II, XZ section shows the orthogonal sections of all the stacks, green- FLAG-hCTR1 and red- F-actin; treatments are on the basolateral chamber of the transwell, scale bar- 5 μm]. **(C)** Immunoblot of FLAG-tagged Wild-type hCTR1 and different mutants probed with α-FLAG-antibody (upper panel). Loading control GAPDH for the respective immunoblots are shown (lower panel).

**Supplementary Table 1.** Primers used in the experiments are listed with names and sequences (5^/^- 3^/^).
